# Dose-ranging pharmacokinetics, safety, and efficacy of a 90-day intravaginal ring delivering islatravir in pigtail macaques

**DOI:** 10.1126/sciadv.aef3514

**Published:** 2026-08-26

**Authors:** Priya Srinivasan, Jasmine L. King, Jining Zhang, Isabella C. Young, Dawn Little, Mackenzie L. Cottrell, Craig Sykes, Amanda P. Schauer, James Mitchell, Angela D. M. Kashuba, J. Gerardo García-Lerma, Soumya Rahima Benhabbour, James M. Smith

**Affiliations:** ^1^ Laboratory Branch, Division of HIV Prevention, Centers for Disease Control and Prevention, Atlanta, GA, USA.; ^2^ Lampe Joint Department of Biomedical Engineering, North Carolina State University and The University of North Carolina at Chapel Hill, Chapel Hill, NC, USA.; ^3^ Division of Pharmacoengineering and Molecular Pharmaceutics, Eshelman School of Pharmacy, University of North Carolina at Chapel Hill, Chapel Hill, NC, USA.; ^4^ Katmai Government Services (KGS), Anchorage, AK, USA.; ^5^ Division of Pharmacotherapy and Experimental Therapeutics, Eshelman School of Pharmacy, University of North Carolina at Chapel Hill, Chapel Hill, NC, USA.

## Abstract

Intravaginal rings (IVRs) are attractive user-controlled delivery systems that can achieve sustained release of compounds for several weeks. We modeled in macaques a three-dimensional (3D)–printed IVR delivering the investigational nucleoside reverse transcriptase translocator inhibitor islatravir (ISL). Three IVRs with 15, 30, or 60 milligrams of ISL were used in 30-day pharmacokinetic and safety studies followed by 90-day studies to identify a dose that maintains the concentration of the active intracellular metabolite islatravir triphosphate (ISL-TP) above protective benchmarks. IVRs were safe and delivered ISL in a dose-dependent manner. In a challenge study involving 12 weekly low-dose vaginal exposures to SHIV162p3, six macaques treated with 60-milligram ISL IVRs for 90 days remained seronegative and SHIV RNA negative, while two untreated controls were infected. These data provide initial preclinical evidence that a 90-day extended-release ISL IVR is safe and offers complete protection in a stringent challenge model, warranting further clinical investigation.

## INTRODUCTION

Globally, there are 40.8 million persons living with HIV (*1*). Women and adolescent girls account for more than half of the persons living with HIV (*1*). Women are disproportionately affected by HIV in many countries (*2*, *3*). Particularly in sub-Saharan Africa, women have a higher risk of HIV infection, accounting for 59% of infections in this region (*4*). Approximately 80% of those infected are adolescent girls aged 15 to 19 years old (*4*). Moreover, evidence suggests that gaps in educational attainment, income, reproductive health, empowerment, and women’s labor market participation play a contributing role to the spread of new HIV cases (*4*). Despite these statistics and decades of research, there are only three Food and Drug Administration (FDA)–approved preexposure prophylaxis (PrEP) products on the market. Until December 2021, tenofovir disoproxil fumarate (TDF) and emtricitabine (FTC) were the only oral antiretroviral (ARV) HIV PrEP therapies available to persons with vaginal exposure to HIV. Cabotegravir long-acting (CAB-LA; Apretude), an HIV-1 integrase strand transfer inhibitor, was the first approved, long-acting injectable HIV PrEP for women and men (*5*). CAB-LA was shown to be superior to daily oral PrEP with individuals 12 times less likely to acquire HIV on Apretude compared to TDF/FTC (Truvada) (*6*). Furthermore, evidence suggests that end-users in target populations of high HIV prevalence have a stronger preference for CAB-LA due to lower user-adherence burden, long-acting duration, and increased user discretion (*7*). In June 2025, the FDA approved a lenacapavir injection (Yeztugo) as the first and only twice-yearly HIV-1 capsid inhibitor for PrEP (*8*). An additional promising long-acting technology for HIV PrEP is the dapivirine (DPV) intravaginal ring (IVR; DapiRing). Although not approved by the FDA, the DPV ring is recommended by the World Health Organization (*9*). Now, there are 11 countries in Africa with approved use for the once-monthly intravaginal DapiRing. Although the landscape of technologies for HIV PrEP is growing, there is still a need for innovative, longer-acting, user-controlled technologies that are highly effective at preventing HIV acquisition for women (*10*).

Globally, ∼50% of all pregnancies are unintended (*11*) with women with HIV having higher rates than uninfected women (*12*, *13*). Because pregnancy prevention largely falls on women, most contraceptive methods (i.e., oral pills, intrauterine devices, implants, and IVRs) are designed for female use. Male and female condoms are the only reversible contraception designed for both male and female end users. Furthermore, condoms are the only approved multipurpose prevention technology (MPT) product to prevent HIV acquisition and unintended pregnancies. Unfortunately, condom use requires male partner cooperation to be an effective prevention method (*14*). Given the co-occurring risks of pregnancy, HIV, and other sexually transmitted infections, an “all-in-one” MPT product could improve women’s sexual and reproductive health.

IVRs are a promising platform that can deliver drugs locally to the female reproductive tract. The benefits of IVRs include women-controlled, discreet, long-acting, controlled release of multiple drugs from a single platform and high acceptability in target populations (*15*–*19*). Now, there are several IVRs on the market globally for contraception (NuvaRing and Annovera) (*15*), hormone replacement therapy (Estring) (*20*), and HIV PrEP (DapiRing) (*9*), highlighting the versatility in the delivery platform to incorporate several active pharmaceutical ingredients (APIs). Now, marketed IVRs use traditional manufacturing processes, such as hot-melt extrusion and injection molding, where the use of high pressure and temperature is inherent to these processes (*21*). Thus, APIs are subjected to high heat and pressures that ultimately limit the number of APIs compatible with this process. Furthermore, traditional IVR manufacturing processes are limited to producing solid cross sections, thus limiting their ability to fine-tune key product attributes (surface area, size, diffusion distances, and compression) that control drug release and end-user preferences. Notably, injection-molded IVRs elicit incomplete drug release, thus requiring manufacturers to overload the device with APIs to achieve therapeutic efficacy (*22*).

Additive manufacturing, or three-dimensional (3D) printing, is compatible with computer-aided design (CAD), which opens the opportunity to easily design IVRs in ways that are not possible with traditional IVR manufacturing. Uniquely implemented IVR designs alter ring surface area by eliminating the solid cross section, provide opportunities to fine-tune and control drug-release kinetics, and can promote complete drug release. The continuous liquid interface production (CLIP) or digital light synthesis (DLS) technology affords the generation of high-resolution smooth and monolithic 3D-printed IVRs through a fast, continuous, layerless, and monolithic process (*23*–*25*).

We used CLIP generated solid silicone-based resin IVRs to establish a safe and effective dose of islatravir (ISL) in pigtail macaques. This will aid with efficient optimization of our intended MPT IVR, combining ISL with two contraceptive hormones (etonogestrel and ethinyl estradiol) (*26*) and generation of geometrically complex IVRs.

Nonhuman primates are regarded as the most pertinent preclinical model for evaluating the safety, pharmacokinetics (PK), and efficacy of HIV PrEP candidates and provide proof of concept for novel HIV intervention strategies (*27*–*29*). The repeat low-dose SHIV162p3 challenge macaque model of PrEP has played a pivotal role in the testing of the effectiveness of various ARVs under conditions that mimic human sexual HIV exposures (*30*, *31*). The nonhuman primate model has been valuable in defining clinically relevant doses for preexposure prophylactic protection against HIV acquisition for a number of ARVs, such as TDF, FTC, CAB-LA, and tenofovir alafenamide (*32*–*34*). Pigtail macaques are considered the gold standard for evaluating intravaginal PrEP modalities as their vaginal physiology and continuous lunar menstrual cycle are like those in women (*35*, *36*). We previously demonstrated the safety, PK, and efficacy of various ARVs in our well-established pigtail model (*37*–*43*). Given the toxicity concerns observed in clinical trials with repeated ISL exposures (reduction in CD4^+^ and lymphocyte counts in peripheral blood) (*44*), we sought to investigate the benchmarks of safety, PK, and efficacy of ISL IVRs by conducting dose-response safety, PK, and long-term (90-day) efficacy studies against repeat SHIV challenges.

Here, we assessed in vitro release and performed a 30-day ISL IVR PK crossover study at ISL doses of 15, 30, and 60 mg in pigtail macaques. Subsequently, we investigated 90-day PK of 15 and 60 mg of ISL IVRs. On the basis of the ISL PK profile over 90 days in vaginal fluids and plasma and ISL–triphosphate (TP) in peripheral blood mononuclear cells (PBMCs) and vaginal tissue, we conducted a 90-day vaginal SHIV challenge study in normal cycling pigtail macaques using 60-mg ISL IVRs. We demonstrate sustained release of ISL at safe and clinically relevant levels of ISL-TP for 90 days and document complete protection against repeated SHIV challenges with a single IVR administration.

## RESULTS

### IVR fabrication with CLIP and postfabrication drug loading

**Fig. 1.**
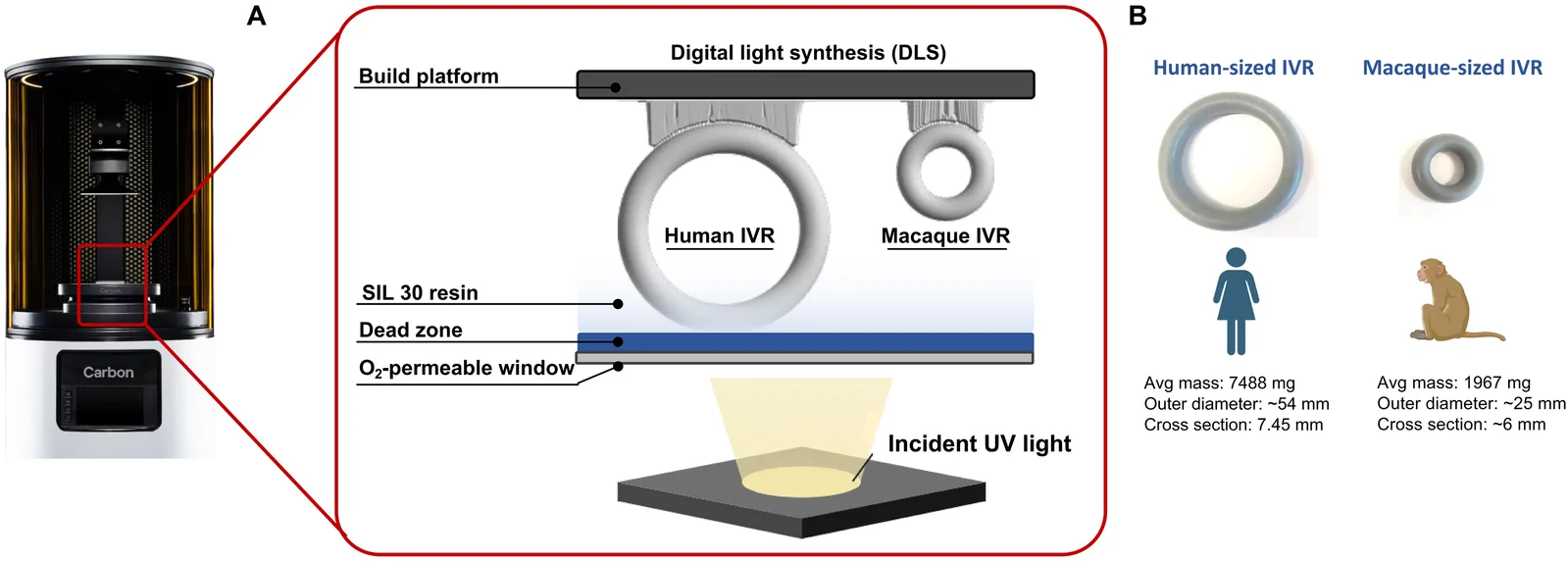
Overview of IVR fabrication. (**A**) Schematic of CLIP 3D printing illustrating CAD designs and fabrication of human- and macaque-sized solid IVRs. UV, ultraviolet. (**B**) Human- and macaque-sized solid IVRs 3D-printed in SIL-30 and their metrics. This figure was partially created in BioRender. Benhabbour, R. (2026) https://BioRender.com/oxks389.

We created human-sized and macaque-sized solid IVRs by using CAD and fabricated with CLIP 3D printing with a two-part silicone (polyurethane) (SIL30, Carbon Inc.) resin as previously reported by Young *et al.* (*26*) (Fig. 1). Briefly, IVRs were fabricated in an M1 3D printer (Carbon) using CLIP, which allows for continuous and layerless fabrication of the IVR. After the ultraviolet cure, IVRs underwent an 8-hour postthermal cure to initiate a secondary reaction, complete the matrix formation, and produce the final IVR.

ISL was incorporated into the IVR postfabrication and postthermal cure via a solvent-mediated postloading process as previously described by Janusziewicz *et al.* (*45*). Drug loading by using this postfabrication loading process allows APIs to be accurately and homogeneously dispersed throughout the matrix. This process is facilitated by the unique material properties of SIL30, allowing the IVRs to swell considerably when placed in a drug-containing organic solvent, referred to as the loading solution. Acetone was selected as the solvent for drug loading because it considerably swells the silicone-based IVR matrix, elicits high drug solubility with ISL, has a low flashpoint, and is classified as a safe solvent (class 3). We developed a weight-based loading approach to achieving target drug loading in our IVRs, referred to as loading equations. Loading equations elicit a linear relationship between drug weight percent [(mass of drug loaded/mass of IVR) × 100)] and the concentration of the loading solution (*26*, *37*). Loading equations for ISL were developed for human-sized IVRs [54-mm outer diameter (OD), 7.6-mm cross section (O’Rourke DM, #25)] and macaque-sized IVRs (25-mm OD, 6.0-mm cross section), to achieve equivalent ISL weight % loading in the human-sized and macaque-sized IVRs.

### In vitro release studies of ISL IVRs

**Fig. 2.**
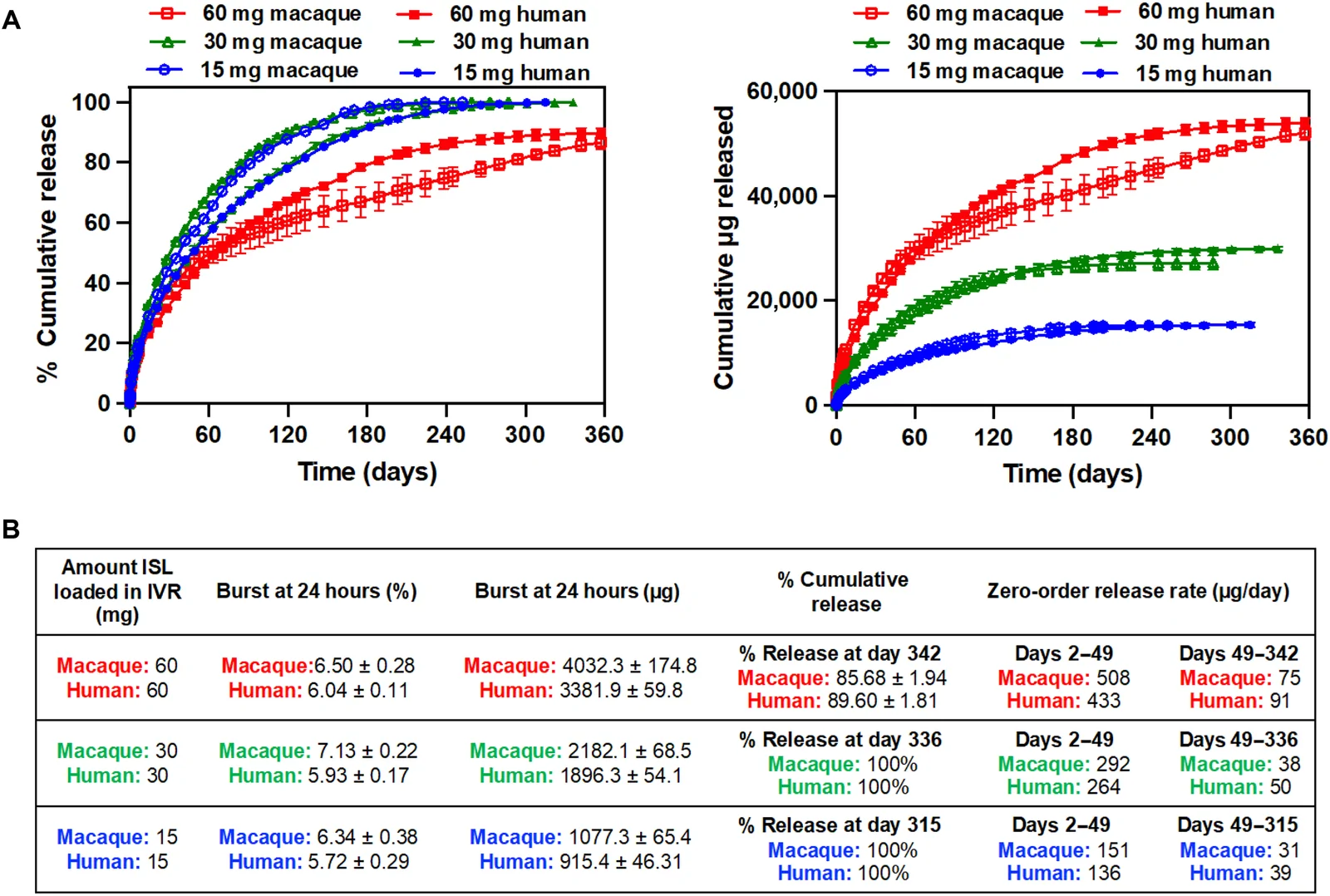
In vitro release of ISL from macaque- and human-sized solid IVRs. (**A**) Cumulative percent (%) and microgram (μg) release of ISL IVRs in SVF (pH 8) at 37°C. (**B**) Summary table of 24-hour ISL burst release, percent release at study completion, and zero-order release rates.

We conducted in vitro release studies to investigate drug release kinetics of ISL from human- and macaque-sized IVRs at ISL doses of 15, 30, and 60 mg. The release of ISL was assessed in simulated vaginal fluid (SVF; 25 mM sodium acetate with 2% Solutol) at 37°C and pH 8 to mimic the macaque vaginal environment. As shown in Fig. 2, ISL elicited a low burst release within the first 24 hours (<7%) from macaque- and human-sized IVRs at all doses. Furthermore, ISL elicited zero-order release kinetics throughout the entire study at all doses. Daily release rates for all ISL doses are summarized in Fig. 2B for macaque- and human-sized solid IVRs. As noted in Fig. 2B, doubling the ISL dose resulted in a twofold increase in release rates within the first 7 weeks of the study. Macaque- and human-sized IVRs reached complete ISL release on days 315 and 336 with the 15- and 30-mg ISL doses, respectively. On the other hand, the 60-mg ISL IVR reached ∼85 to 90% ISL release at day 360. Collectively, these results demonstrate sustained release of ISL for at least 90 days in macaque vaginal pH with all three ISL doses investigated.

### In vivo 28-day dose-response PK study in pigtail macaques

**Fig. 3.**
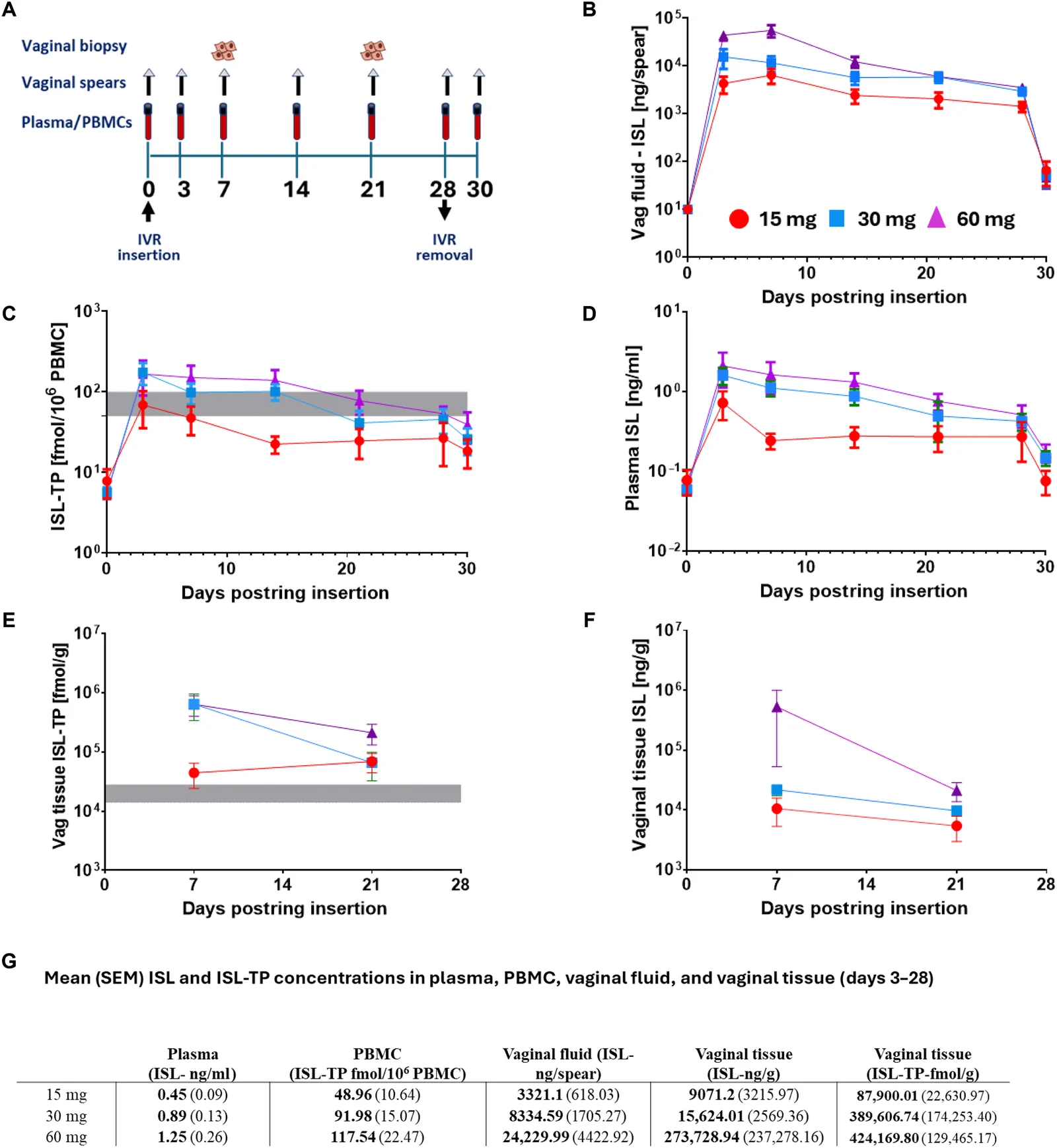
Macaque 28-day PK of ISL IVR. (**A**) Macaque 28-day PK study design. Mean and standard error of the mean (SEM) drug concentrations (*n* = 8 macaques) of ISL and ISL-TP in (**B**) vaginal fluid (nanograms per spear), (**C**) PBMCs (femtomoles per 10^6^ PBMCs), (**D**) plasma (nanograms per milliliter), (**E**) vaginal tissue (femtomoles per gram), and (**F**) vaginal tissue (nanograms per gram). (**G**) Summary table of mean (SEM) ISL and ISL-TP concentrations in the various matrices between days 3 and 28. Red circle, blue square, and purple triangle represent 15-, 30-, and 60-mg ISL IVR, respectively. All animals received all three doses for 28 days with a drug washout period of 2 or more weeks between applications. The gray bar in (C) denotes PK benchmark of 50 to 100 fmol/10^6^ PBMCs and (E) converted to femtomoles per gram of tissue based on a conversion factor of 284,355 cells/mg of tissue. Individual macaque values for plasma, PBMCs, vaginal tissue, and fluids are shown in fig. S2.

On the basis of our previous macaque work with ISL IVRs (*26*) and the promising in vitro release data obtained with the 15-, 30-, and 60-mg IVRs in this study, we evaluated the PK in a series of 28-day cross-over PK studies in pigtail macaques (*n* = 8 for each dose; Fig. 3A and fig. S1) and determined ISL concentrations in different matrices. Plasma, vaginal fluids, vaginal tissue, and PBMCs were collected at prespecified time points for ISL and ISL-TP quantitation (*46*, *47*). Figure 3 (B to G) represents the drug concentrations detected in the different matrices throughout the studies. Throughout all compartments, ISL concentrations were sustained for 28 days with the 30- and 60-mg ISL IVR. Mean ISL-TP levels in PBMCs at or above the established PK benchmark for oral dosing (50 to 100 fmol/10^6^) (*48*) were achieved with the 60-mg IVRs, while it was only seen until day 14 for the 30-mg IVRs. With the 15-mg IVRs, mean ISL-TP concentrations were at the target level only during the burst release on day 3. Plasma ISL concentrations with the 15-, 30-, and 60-mg IVRs were within the levels seen with the safe, 0.25-mg once-daily oral ISL human dose (*C*_avg_ geometric mean—0.21 ng/ml) (*49*). As seen in Fig. 3 (B and G), in vaginal fluids, the mean ISL concentrations were dose proportional with the 30- and 60-mg IVR levels being threefold and sevenfold greater than the 15-mg IVR, respectively. The concentration with the 60-mg IVR was threefold greater than that seen with the 30-mg IVR. Likewise, a dose proportionality was seen in both vaginal tissue ISL and ISL-TP concentrations. The mean ISL concentrations in vaginal tissue with 30 and 60 mg were 2-fold and 30-fold greater than the 15-mg IVR, respectively. The concentration with the 60-mg IVR was 18-fold greater than that seen with the 30-mg IVR. The mean ISL-TP concentration in vaginal tissue with the 30- and 60-mg IVRs were fourfold and fivefold greater than the 15-mg IVR, respectively, while it was similar between the 30- and 60-mg IVRs. Overall, the PK profiles over 28 days elicited a dose response that corresponded with the drug loading in the rings. Figure S2 shows individual macaque drug concentrations in plasma, PBMCs, vaginal fluids collected proximal and distal to the IVR (fig. S2, A and B), and vaginal tissue (fig. S2, C and D) with 15-, 30-, and 60-mg IVRs. These plots illustrate that variability in ISL and ISL-TP is much greater in the lower doses than with the 60-mg IVR. Table S1 shows the median (range) ISL and ISL-TP concentrations obtained in the different matrices, while the 15-, 30-, and 60-mg IVRs were present in the macaques for 28 days. Plasma ISL and PBMC ISL-TP concentrations were below the limit of quantification (LOQ) at all time points throughout the study in two of the eight macaques that received the lower 15-mg dose. Quantification of residual drug upon retrieval of IVRs from the macaques showed that 57.4, 54.1, and 50.4% ISL were released from the 15-, 30-, and 60-mg IVRs, respectively, after 28 days (Table 1). At 2 days post-IVR removal, there was a fivefold, fivefold, and sixfold decrease in ISL in plasma and a twofold, threefold, and threefold decrease ISL-TP in PBMCs for the 15-, 30-, and 60-mg ISL IVRs, respectively. In addition, there was a 14-fold, 71-fold, and 229-fold ISL decrease in vaginal fluids for the 15-, 30-, and 60-mg ISL IVRs, respectively (Fig. 3). No vaginal biopsies were collected postring removal to determine if vaginal tissue may retain drug after IVR removal.

**Table 1.** Percent (%) ISL released calculated from residual drug measurements in used IVRs. IVRs were extracted ex vivo, and residual ISL was quantified by HPLC analysis.

Study period	Dose (mg)	Percentage of ISL released median (range)
30-day PK	15	57.4 (48.9–73.7; *n* = 7)
	30	54.1 (52.9–69.8; *n* = 8)
	60	50.4 (46.3–70.2; *n* = 8)
90-day PK	15	93.8 (92.4–94.0; *n* = 3)
	60	56.1 (49.1–59.6; *n* = 3)
90-day efficacy	60	64.9 (53.1–69.6; *n* = 6)

We assessed the in vivo vaginal safety of the 15-, 30-, and 60-mg ISL IVRs throughout the study by monitoring vaginal pH and systemic CD4 and CD8 frequencies, as well as visual observations to monitor for tissue irritation. All macaques maintained vaginal pH within the normal range (6.5 to 8.7) for pigtail macaques (fig. S3). The median pH decrease when the ring was present through day 28 for the 15-mg ISL IVR relative to baseline pH (day 0: 8.3, days 3 to 28: 7.7) and the 30-mg ISL IVR (day 0: 8.2, days 3 to 28: 7.1) is similar to studies previously reported with other IVRs (*27*, *50*, *51*). The median vaginal pH for the 60-mg ISL IVR was unchanged (day 0: 7.1, days 3 to 28:7.1). Given the dose-dependent decreases in CD4 and lymphocyte count seen in clinical trials with oral ISL (*52*), we monitored CD4 and CD8 frequencies throughout the study. CD4^+^ and CD8^+^ T cell frequencies were monitored for all animals throughout the extensive crossover PK study (fig. S4). CD4 and CD8 cell frequencies did not show any statistically significant differences (Kruskal-Wallis test, *P* > 0.05) with all ISL doses for 28 days when compared to baseline (day 0) and postring removal (day 30). No visual signs of tissue irritation were observed while the rings were present during the entire study period.

### In vivo 90-day PK study in pigtail macaques

**Fig. 4.**
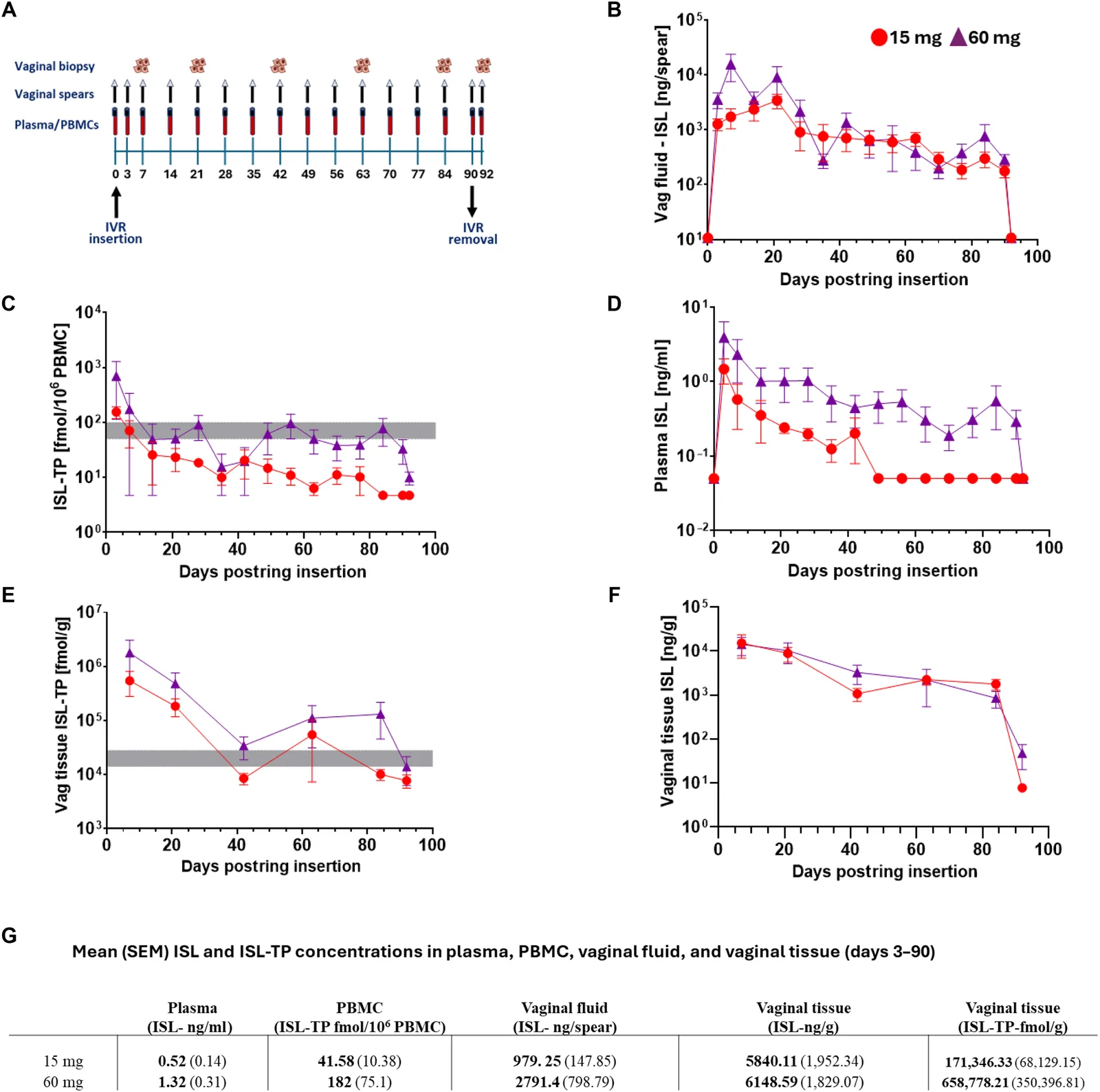
Macaque 90-day PK of ISL IVRs. (**A**) Macaque 90-day PK study design. Mean and SEM drug concentrations (*n* = 3 macaques) of ISL and ISL-TP in (**B**) vaginal fluid (nanograms per spear), (**C**) PBMCs (femtomoles per 10^6^ PBMCs), (**D**) plasma (nanograms per milliliter), (**E**) vaginal tissue (femtomoles per gram), and (**F**) vaginal tissue (nanograms per gram). (**G**) Summary table of mean (SEM) ISL and ISL-TP concentrations in the various matrices between days 3 and 90. Red circle and purple triangle represent 15- and 60-mg ISL IVRs, respectively. The gray bar in (C) denotes the PK benchmark of 50 to 100 fmol/10^6^ PBMCs and (E) converted to femtomoles per gram of tissue based on a conversion factor of 284,355 cells/mg of tissue. Individual macaque values for plasma, PBMCs, vaginal tissue, and fluids are shown in fig. S5.

Given that ISL has not been previously delivered topically, the main objective of the present work was to establish a benchmark of efficacy for our 90-day ISL IVR. Hence and on the basis of results from the 28-day dose-response PK study, we subsequently conducted an extended 90-day PK study with the low-dose (15 mg) and high-dose (60 mg) ISL IVRs in pigtail macaques (*n* = 3 each) as illustrated in Fig. 4A. We collected plasma, PBMCs, vaginal fluid, and vaginal tissue at predetermined time points throughout the study to quantify ISL and ISL-TP concentrations (Fig. 4, B to F). Throughout all compartments, ISL concentrations were maintained generally for 90 days with the 60-mg ISL IVR in two of the three macaques. ISL-TP concentrations were at or above the established PK benchmark for oral dosing of 50 to 100 fmol/10^6^ PBMCs in 74% of the samples while the ring was present in the above two macaques (*48*). Both plasma ISL and ISL-TP were below the limit of detection (LOD) throughout the study in one macaque that received the 60-mg IVR, while ISL was detectable both in vaginal fluid and tissue. Plasma ISL concentrations fell below LOQ after day 42 in all three macaques that received the 15-mg ISL IVRs. As seen in Fig. 4 and summary Table 4G, in vaginal fluids, the mean ISL concentration was dose proportional with the 60-mg IVR levels being threefold greater than the 15-mg IVR. While the mean concentration of ISL detected in the vaginal tissue was similar between the 15- and 60-mg IVRs, the ISL-TP concentration was fourfold greater in the 60-mg IVR than the 15-mg IVR. At 2 days post-IVR removal, there was a 5-fold and 13-fold decrease in ISL in plasma and a 4-fold and 15-fold decrease ISL-TP in PBMCs for the 15- and 60-mg ISL IVRs, respectively. In addition, there was a 46-fold and 130-fold ISL decrease in vaginal fluids for the 15- and 60-mg ISL IVRs, respectively, and a 622-fold and 110-fold ISL decrease in vaginal tissue for the 15- and 60-mg ISL IVRs, respectively, 2 days post-IVR removal. There was a threefold and fourfold ISL-TP decrease in vaginal tissue for the 15- and 60-mg ISL IVRs, respectively, 2 days post-IVR removal. Quantification of residual drug in IVRs ex vivo showed that 93.8 and 56.1% ISL was released from the 15- and 60-mg IVRs, respectively, after 90 days in macaques (Table 1). Individual macaque’s drug concentrations in plasma, PBMCs, vaginal fluids collected proximal and distal to the IVR, and vaginal tissue are summarized in fig. S5 for both the 15- and 60-mg ISL IVRs. Table S2 shows the median (range) ISL and ISL-TP concentrations obtained in all matrices over 90 days of use of the 15- and 60-mg ISL IVRs.

### Safety of ISL IVR over 90 days

We evaluated the in vivo vaginal safety of the 15- and 60-mg ISL IVRs over 90 days by monitoring plasma progesterone and systemic CD4 and CD8 frequencies. No abnormal variation was observed in the plasma progesterone cycling patterns of the macaques in the presence of the IVR for 90 days. As with the 30-day PK studies, we monitored CD4 and CD8 frequencies in PBMCs collected longitudinally throughout the 90 days while the 15- and 60-mg ISL IVRs were present (fig. S6). The Kruskal-Wallis test of the CD4 (15-mg IVR, *P* = 0.77; 60-mg IVR, *P* = 0.87) and CD8 (15-mg IVR, *P* = 0.4; 60-mg IVR, *P* = 0.78) cell frequencies did not show any statistically significant differences with either of the 15- or 60-mg ISL IVRs over 90 days in comparison to the baseline (day 0) and postring removal (day 92).

### Complete protection by 60-mg ISL IVR against weekly vaginal SHIV162p3 challenges

**Fig. 5.**
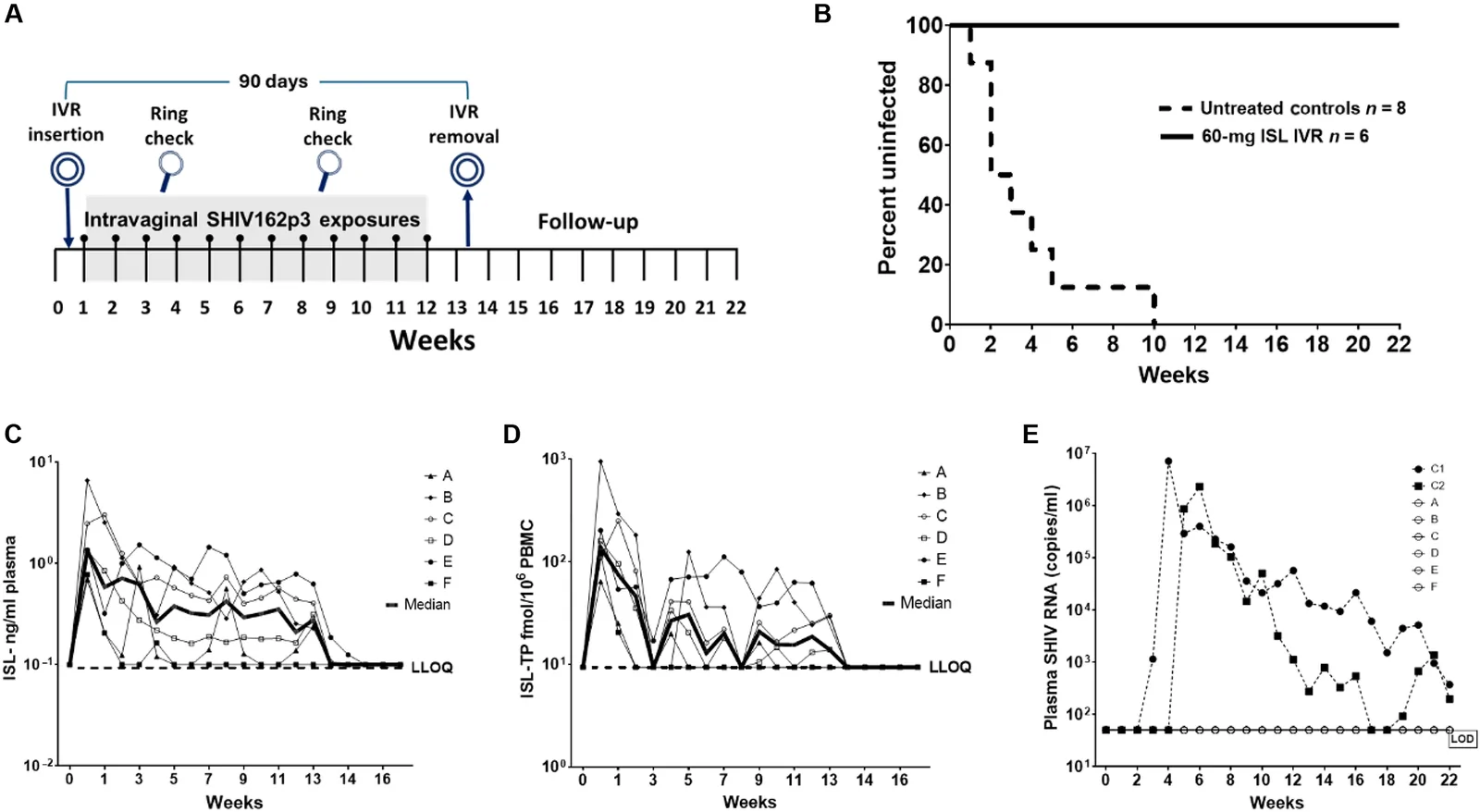
Efficacy of PrEP with a 90-day 60-mg ISL IVR. Six normally cycling pigtail macaques (designated A to F) received 60-mg ISL IVR 4 days after baseline and were exposed to weekly vaginal SHIV162p3 challenges starting at day 7 for 12 weeks. Eight additional animals were used as controls [two real-time (C1 and C2) and six historical controls]. (**A**) Macaque efficacy study design, (**B**) Kaplan-Meier plot for time to infection relative to the study period, (**C**) ISL and (**D**) ISL-TP levels in plasma and PBMCs between weeks 0 and 17 in the six macaques that received the 60-mg ISL IVR. The bold black line represents the medium drug concentrations. (**E**) SHIV RNA levels in plasma. LLOQ, lower limit of quantification.

On the basis of results from the 90-day PK study, we selected the 60-mg ISL IVR to assess efficacy in preventing vaginal SHIV infection. Figure 5A illustrates the 22-week efficacy study design. The 60-mg ISL IVRs were inserted into six normally cycling pigtail macaques 3 days before the first vaginal SHIV162p3 exposure and remained in place for 90 days (13 weeks). All macaques were exposed to up to 12 weekly atraumatic vaginal SHIV162p3 challenges. Macaques were followed for an additional 9 weeks after ring removal to detect potential late breakthrough infections due to viral suppression of remote reservoirs during IVR use. Blood was collected weekly throughout the study for plasma viremia detection, ISL and ISL-TP drug quantitation, T cell frequencies, and seroconversion. Figure 5B shows infection outcome in macaques that received 60-mg ISL IVR and were challenged weekly for 3 months in comparison to two real-time and six contemporary controls exposed to the same virus stock, dose, and inoculation method (*53*).The two real-time controls were infected at challenges 2 and 4, and historical controls were infected at challenges 2, 2, 1, 3, 5, and 10. Overall, untreated animals became infected after a median of 2.5 challenges (range 1 to 10). In contrast, all six macaques that received the ISL IVR were uninfected after 12 SHIV exposures and remained uninfected and seronegative throughout the 10-week follow-up period (*P* = 0.0003, Mantel-Cox test). The estimated efficacy of the 60-mg ISL IVR was 100% (95% confidence interval = undefined). Figure 5C represents the plasma ISL and PBMCs ISL-TP, respectively, that were seen in the IVR macaques during the first 18 weeks of the study. Median plasma ISL concentration while the ring was present for 13 weeks was 0.42 ng/ml (0.1 to 6.59 ng/ml) and similar to those observed in the 30- and 90-day PK studies. The median ISL-TP concentration was 20.5 (9.38 to 954) fmol/10^6^ cells and was lower than the concentrations observed in the 30- and 90-day PK studies (63.62 and 82.18 fmol/10^6^ cells, respectively). As was seen in the PK studies, variability in drug levels was observed in plasma and PBMCs with two of the six macaques having ISL-TP concentrations that were below LOQ after week 1 (Fig. 5C). Both ISL and ISL-TP became undetectable following ring removal. Quantification of residual drug in IVRs ex vivo showed that 35.1% ISL remained after 90 days in macaques corresponding to a median (range) release from the 60-mg IVRs of 64.9% (53.1 to 69.6%) over 90 days (Table 1). The real-time controls exhibited typical plasma viremia (Fig. 5D) and seroconverted 2 to 3 weeks after infection. The late luteal/early follicular phase has been reported to be the window of highest susceptibility to vaginal SHIV infection in cycling pigtail macaques (*54*, *55*). Five of the six macaques had received virus exposures over at least three full menstrual cycles (fig. S7). For macaque D, the progesterone levels indicate that the macaque was not cycling until week 12 (fig. S7).

The presence of the 60-mg IVR for 90 days did not cause any statistically significant differences (fig. S6; Kruskal-Wallis with Dunn’s multiple comparison test, *P* > 0.05 for each of the 12 tests) in the CD4 and CD8 frequencies in comparison to the baseline (week 0) and post-IVR removal (weeks 14 to 22). This analysis was carried out for each animal individually (fig. S8). The untreated controls (C1 and C2) showed a decline in the CD4 T cell frequencies during the study (fig. S8).

## DISCUSSION

We have evaluated the safety, PK, and efficacy of a 3D-printed IVR releasing ISL in a pigtail macaque model. We systematically assessed rings containing different ISL doses to identify a dose that is safe and can release ISL above protective benchmarks for 90 days (*26*). The ISL IVRs were found to be safe in macaques in all PK studies, and sufficient ISL was released from a single 3D-printed 60-mg ISL IVR for 90 days resulting in complete protection in macaques from 12 weekly virus exposures. To our knowledge, this is the first evaluation of an IVR delivering ISL in this challenge model for 90 days with a single administration and without exchanging rings every 30 days.

Topical products for HIV PrEP are viable options for individuals who do not choose daily oral regimens or long-acting injectables. Topical products offer a discreet and user-controlled option for young girls and women, can deliver high drug doses locally at the point of viral entry, and generally result in lower systemic exposure to drugs. PK benchmarks for vaginal sustained-release topical products, especially vaginal rings, are typically restricted to local fluids and tissue levels, because with most IVR studies, there is only sporadic detection of ARVs in plasma (*56*). However, ISL differs from other ARVs as consistent systemic exposure was observed when ISL was released from our IVRs. This allowed for easier monitoring of ISL in plasma as well as ISL-TP in PBMCs. Because the protective threshold for ISL from rings in macaques is not known, we set our target levels for ISL-TP at 50 to 100 fmol/10^6^ PBMCs to match protective benchmarks following oral administration (*48*). The reasons for durable ISL exposures in blood following vaginal IVR application may be related to continuous absorption of ISL from the vaginal mucosa and slow drug clearance from plasma due to a long half-life in plasma of 41 hours (*57*). The importance of systemic exposure to ISL and its impact on protection are unclear, but having an extra layer of defense beyond the mucosal point of entry should be considered beneficial.

The development of ISL for oral PrEP experienced setbacks in late 2021 when several trials were paused due to exposure-related declines in total lymphocyte counts (*44*). We show here no effect of ISL on CD4 or CD8 T cells, suggesting that topical exposure to ISL may help mitigate these clinical toxicities. We also found that some of the animals were protected even with ISL-TP concentrations in PBMCs below the protection benchmark of 50 fmol/10^6^ cells. These findings underscore the importance of tissue drug concentrations in providing protection and suggest that protection benchmarks may vary between oral and topical products.

We used three doses in the 30-day PK crossover study design to obtain data from eight animals for each dose. A similar release profile was observed in all matrices between the 30- and 60-mg ISL rings with ISL-TP in PBMCs reaching concentrations well above the 50- to 100-fmol target in some of the macaques and a clear reduction in ISL and ISL-TP concentrations with the 15-mg dose in three of the eight macaques. All three doses resulted in sustained ISL concentrations in all matrices for 28 days, and local ISL concentrations in vaginal fluids decreased by ∼100-fold 2 days after IVR removal. ISL-TP in PBMCs and ISL in plasma did not decrease as markedly (three- and sixfold, respectively), suggesting that systemic concentrations are maintained and may provide some protection even after ring removal. Vaginal tissue levels of ISL and ISL-TP were high with all three doses with ISL-TP being well above the estimated target concentration of 14,000 to 28,000 fmol/g of tissue at days 7 and 21 when biopsies were taken (*26*).

Our goal for this ISL IVR formulation was to evaluate efficacy from multiple weekly virus exposures with a single 90-day formulation, which would remove any variability inherent in multiple ring exchanges and offer more infrequent dosing if advanced for human use. We chose to conduct the 90-day PK study with the 15- and 60-mg formulations because we observed only minor differences in the 30-day PK profiles of the 30- and 60-mg ISL IVRs. Differences between doses were most apparent in the systemic ISL in plasma and ISL-TP concentrations in PBMCs with the 60-mg ISL IVR, which reached higher levels in the first 30 days as observed in the 28-day PK studies. The systemic concentrations from day 30 to day 90 were vastly higher with the 60-mg ISL IVR compared to the 15-mg ISL IVR where ISL-TP became undetectable at several late time points and plasma ISL became undetectable in all samples from day 49 to day 90. As expected, the 15-mg ISL IVR released most of its ISL (93.8%) in 90 days, while the 60-mg IVR still had 44% of ISL remaining after 90 days. Additional studies are needed to understand whether the 60-mg IVR continues to release ISL above the protection threshold beyond 3 months. Unexpectedly, there was little difference in vaginal ISL and ISL-TP concentrations between the two doses in the later weeks in both fluids and tissues.

The repeat exposure pigtail macaque model has been used to evaluate numerous vaginal PrEP products, including gels, inserts, and vaginal rings, and is considered the best model available for preclinical evaluation (*42*, *43*, *58*–*63*). The dose of virus chosen has consistently resulted in controls being infected in a reasonable time frame and number of exposures in naturally cycling female macaques (*26*). All six ISL IVR–treated macaques were protected from 12 weekly challenges spanning roughly three menstrual cycles with a single application. Long-acting products, such as cabotegravir (CAB-LA) for HIV PrEP, have gained increased acceptability in recent years, and the newest option, lenacapavir, has been shown to be highly protective in women (*64*). Sustained release of ISL from a 90-day IVR would require administration only four times per year and may be preferable for some populations by giving the option to the patient to remove the ring and therefore the drug at any time. A 90-day ISL IVR for HIV PrEP may improve adherence by reducing the number of IVRs to only four (*4*) rings per year compared to 12 monthly DapiRings. Future iterations as an MPT IVR by adding contraceptive hormones will also benefit from a 90-day application (*26*). We recently showed in sheep that a similar human-sized 3D-printed IVR was safe and delivered sustained concentrations of ISL and the hormonal contraceptives etonogestrel and ethinyl estradiol (*26*).

There are limitations to our study. This proof-of-concept study was conducted with only ISL in the IVR, leaving out the hormonal contraceptive components. This decision was made because of a lack of available experimental data on the level of ISL needed with a topical product to protect pigtail macaques from vaginal SHIV infection. We can conclude that the levels observed in this study were sufficient for protection, but further studies are needed to determine the lower limit of ISL required for protection or if the addition of a hormonal contraceptive would affect efficacy. Our study was also not designed to identify a protection threshold for topically applied ISL. For instance, we do not know whether residual ISL-TP remaining in tissues after IVR removal will continue to provide some levels of protection and allow for some product forgiveness.

In summary, we have shown that a 3D-printed ISL IVR has favorable PK and safety profiles in macaques and confers complete protection against repeated virus exposures spanning 3 months with a single application. Together with the safety studies conducted in sheep, we feel that this product may offer an attractive alternative MPT product for women who choose to not use long-acting injectables or on-demand topical products, and if performance and safety are demonstrated in women, it could expand preventive options for women and adolescent girls around the world, particularly in areas where HIV is most prevalent. Moreover, the 3D-printed IVR platform can potentially be adapted to accommodate other ARVs and hormonal contraceptives in a single multipurpose prevention option.

## MATERIALS AND METHODS

### Materials

Siliconeurethane-based resin (SIL30) was supplied by Carbon Inc. Phosphate-buffered saline (pH 7.4), hydrochloric acid solution (2 N), high-performance liquid chromatography (HPLC) grade water with 0.1% trifluoroacetic acid (TFA), acetonitrile (ACN) with 0.1% TFA, and acetone were purchased from Thermo Fisher Scientific (Waltham, MA, USA). Kolliphor-HS 15 (Solutol-HS 15) and sodium acetate were purchased from Sigma-Aldrich (St. Louis, MO, USA). ISL was purchased from Synnovator Inc. [Chemical Abstract Service (CAS) # 865363-93; Cary, NC, USA].

### CAD of macaque-sized solid IVRs

Solid IVRs were generated by using CAD in SolidWorks (Dassault Systèmes) and subsequently converted to a standard tessellation language (.STL) file as previously reported (*26*, *65*). Briefly, the .STL file was imported into Magics (Materialise) with the selected unit cell arrayed into a template. Rings were exported as .STL file on the Carbon user interface (UI) for fabrication. Macaque-sized IVR dimensions were noted as 25-mm OD and 6-mm cross-sectional diameter (CSD). Human-sized IVR dimensions were noted as 54-mm OD and 7.6-mm CSD.

### Fabrication of CLIP 3D-printed IVRs

Solid IVRs (macaque-sized and human-sized) were fabricated by using SIL30 resin on an M1 DLS 3D printer (Carbon Inc.). IVRs were oriented vertically to support on the build platform on the Carbon UI. The two-part SIL30 resin was mixed by using a static mixer and added to the M1 cassette. For human-sized IVRs, ∼190 g of SIL30 resin was dispensed for vertical printing of 16 solid IVRs. For macaque-sized IVRs, ∼130 g of SIL30 resin was dispensed for vertical printing of 40 solid IVRs. Fabrication time for human-sized IVRs was ∼3 hours and 6 min and 1 hour and 37 min for macaque-sized IVRs. After 3D printing fabrication, IVRs were removed from the build platform, supports were removed, and rings were smoothed by using a smoothing tool. Solubilized resin was removed from IVRs by washing with isopropyl alcohol for 2.5 min. IVRs were subsequently air-dried for 45 min and placed in a programmable oven for 8 hours at 120° C to allow secondary thermal curing process, as previously reported (*24*, *45*).

### Reverse-phase HPLC analysis

Reverse-phase HPLC analysis was done on an Agilent 1260 II HPLC System (Agilent Technologies, Santa Clara, CA, USA) with a diode array detector and liquid chromatography (LC) pump with autosampler. The stationary phase used for the analysis of ISL was an Inertsil ODS-3 column (4.6 mm by 150 mm, 5 μm) maintained at 40°C. A gradient method was used to achieve chromatographic separation by using 95% of 0.1% TFA in water and 5% of ACN mobile phase. A flow rate of 1.0 ml/min was used with a total run time of 25 min for each 25-μl sample injection. ISL was read at a retention time of 5.9 min and a wavelength of 265 nm.

### Postfabrication drug loading

ISL was loaded into the 3D-printed SIL30 IVR matrix using a postfabrication swelling method as previously reported (*26*). Briefly, IVRs were placed in acetone containing ISL, referred to as drug-loading solution, resulting in drug uptake into the IVR matrix via absorption. Acetone was selected for its ability to swell the silicone-based IVR matrix, being highly solubilizing for ISL (23 mg/ml), as highly volatile and easily removed during IVR drying step, and as a class 3 solvent (low toxicity and low risk to human health). A weight-based loading approach was used, referred to as loading equation, to achieve target loading of ISL into the IVR. The loading equations for ISL were developed and validated for human and macaque-sized IVRs and elicited a linear relationship between drug weight percent [(mass of drug/mass of IVR) × 100] and the concentration of the loading solution. Loading equations were developed by incubating rings in a series of drug concentrated acetone solutions (*n* = 3 IVRs per concentration). Concentrations were determined to ensure that target drug loading would fit within the linear range of the output loading equation. ISL concentration in the loading solutions was quantified by HPLC analysis. IVRs were submerged in the loading solution for 24 hours to reach equilibrium swelling and subsequently air dried overnight followed by drying in an oven under vacuum pressure at 37°C for 48 hours to facilitate acetone removal. To quantify drug loading, dried IVRs were individually submerged in acetone for 48 hours to facilitate drug extraction. Extraction solutions were quantified by HPLC to determine the amount of drug loaded onto each IVR. Loading equations were generated by plotting the concentration of the loading solutions on the *x* axis and the weight % [(mass of drug extracted from IVR/initial mass of IVR) × 100] on the *y* axis.

To load the target amount of ISL into SIL30 matrix (60 mg per IVR), IVRs were incubated in acetone containing ISL (2.57 mg/ml) for 24 hours and subsequently dried for 48 hours to fully remove acetone. To confirm drug loading, dried IVRs were cut into four equal pieces and placed in fresh acetone for 48 hours to completely extract ISL from the IVR. Extraction solution was analyzed by HPLC to determine the amount of ISL loaded into the IVR.

Human-sized IVRs for in vitro release studies were loaded in a single step with 15-, 30-, or 60-mg ISL using the established loading equation. To accomplish this target loading, solid IVRs of known masses were placed in drug-loaded acetone solution [ISL (0.64, 1.28, and 2.57 mg/ml); 100 ml per IVR] for 24 hours to facilitate drug uptake and yield IVRs loaded with 15-, 30-, or 60-mg ISL, respectively. IVRs were removed from drug concentrated solution and dried using the aforementioned method. During the drying process, IVRs were massed daily, and drying was considered complete once the mass of IVR reached equilibrium. After drying, *n* = 1 IVR was extracted in 150 ml of acetone for 48 hours to confirm and validate drug loading. Extraction solution was diluted with ACN and quantified on HPLC.

### Dose-ranging cumulative in vitro release studies

In vitro release kinetics were assessed with human- and macaque-sized IVRs loaded with 15-, 30-, or 60-mg of ISL. IVRs were placed in SVF at pH 4.2 (human vaginal pH). Drug-loaded macaque-sized IVRs were placed in SVF (pH 8) [macaque vaginal pH (*27*, *35*) in a shaking incubator under dynamic conditions (120 rpm)] and incubated at 37°C. The SVF was adapted from Owen and Katz (*66*) and consisted of 25 mM sodium acetate buffer adjusted to pH 8 (macaque vaginal pH); 2% Solutol was added to maintain sink conditions. Macaque-sized IVRs were released in 100 ml of SVF, and human-sized IVRs were released in 200 ml of SVF. Sample aliquots (1 ml) were collected daily for the first 7 days weekly thereafter, and drug concentration was analyzed by HPLC. The media was replaced on days 1 and 3 and weekly thereafter for the duration of the study. ISL concentration was determined by HPLC analysis with a method developed and validated to analyze ISL. Cumulative ISL release was calculated from the HPLC analysis and was normalized to the total mass of drug in the IVR. All experiments were done in triplicate.

### Macaques and virus stock

Female pigtail macaques used in the PK and efficacy studies (*n* = 10, age 6 to 11 years, 5.5 to 9.4 kg) were housed in a United States Department of Agriculture–registered, Office of Laboratory Animal Welfare–assured Association for Assessment and Accreditation of Laboratory Animal Care (AAALAC)–accredited facility with husbandry and enrichment under the care of Centers for Disease Control and Prevention (CDC) veterinarians. All macaque procedures were done under a CDC Institutional Animal Care and Use Committee–approved protocol (protocol nos. 3196SMIMONC and 3224SMIMONC) in accordance with the Guide for the Care and Use of Laboratory Animals (*67*). This activity was reviewed by CDC, deemed research not involving human subjects, and was conducted consistent with applicable federal law and CDC policy. Macaques were sedated with ketamine (10 mg/kg) before all sample collections. SHIV162p3, a chimeric SIVmac239 that contains an HIV-1 subtype B CCR5–tropic envelope, was obtained from the National Institutes of Health Reagent Program (catalog no. ARP-6256). Challenge stocks were prepared and titered in pigtail PBMCs and then stored as individual 1-ml aliquots of 50 median tissue culture infectious dose until thawed before weekly vaginal challenges as described previously (*58*).

### PK and safety studies

IVRs containing 15-, 30-, and 60-mg ISL were inserted with the aid of a pediatric speculum and forceps on day 0 into pigtail macaques (*n* = 8) in a crossover design (fig. S1) and remained in place for 28 days. The sample collections were carried out as per previously established protocols (*26*, *37*, *41*). Blood obtained throughout the study was processed to yield plasma and PBMCs. Weekly plasma progesterone levels were measured with an enzyme immunoassay at the University of Wisconsin National Primate Research Center (*68*). Vaginal fluid proximal and distal to the IVR was collected by using merocel spears (Beaver-Vistec International, Waltham, MA) on days 0 (before ring insertion), 3, 7, 14, 21, 28, and 30 (2 days postring removal) for drug analysis. Changes in vaginal pH were measured by rolling a Dacron swab with vaginal fluid on a pH colorimetric indicator strip (Millipore, Billerica, MA). Vaginal pinch biopsies were collected on days 7 and 21 proximal and distal to the ring by using a Miltex Townsend model 30-1445 minibite cervical biopsy punch forceps.

The 15- and 60-mg ISL IVRs were subsequently investigated for 90-day PK studies (*n* = 3 each). The sample collection was performed as described in Fig. 4. The IVRs were inserted as described above on day 0 and remained in place for 90 days. Blood and vaginal fluids were collected throughout the study (days 0, 3, 7, 14, 21, 28, 35, 42, 49, 56, 63, 70, 77, 84, 90, and postring removal on day 92). Vaginal pinch biopsies were collected on days 7, 21, 42, 63, 84, and 92 weighed, frozen on dry ice, and stored at −80°C.

Multicolor flow cytometry analysis was performed on PBMCs. The PBMCs were incubated with the following monoclonal antibodies (clone numbers in parentheses): CD20-FITC (2H7), CD3-Alexa 700 (SP34-2), CD8-V450 (RPA-T8), and CD4-V500 (L200) for 20 min at 4°C (BD Biosciences, San Jose, CA). The cells were then washed and fixed with 1% paraformaldehyde, and 100,000 events per sample were acquired on BD FACSymphony (BD BioSciences, San Jose, CA). The data were then analyzed on FlowJo software, v10 (Tree Star, San Carlos, CA).

### Analytical methods of macaque PK samples

All drug concentrations were quantified at the University of North Carolina Center for AIDS Research Clinical Pharmacology and Analytical Chemistry Core by using liquid chromatography–tandem mass spectrometry methods

### Drug quantification in pigtail macaque blood

Plasma was separated from EDTA-treated whole blood by centrifugation. Following protein precipitation with the isotopically labeled internal standard (^13^C^15^N_3_-ISL), analytes were separated by using reverse-phase chromatography via a Waters Atlantis T3 (2.1 mm by 50 mm, 3.0-μm particle size). An AB Sciex API-5000 triple quadruple mass spectrometer was used to detect analytes and internal standards under positive ion electrospray conditions. Precision and accuracy of the calibration standards and quality control samples were within 15% for the following dynamic range: 0.1 to 100 ng/ml of ISL.

PBMCs were isolated from EDTA-treated whole blood, counted, and then lysed in 70:30 methanol:water. Following protein precipitation extraction, the phosphorylated active metabolite (ISL-TP) was separated by using anion exchange chromatography via a Thermo Biobasic AX (2.1 mm by 50 mm, 5-μm particle size) analytical column. An AB Sciex API-5000 triple quadruple mass spectrometer was used to detect ISL-TP and internal standard [^13^C_9_,^15^N_3_–2′-deoxyadenosine 5′-triphosphate (dATP)] under positive ion electrospray conditions. Precision and accuracy of the calibration standards and quality control samples were within 15% for a dynamic range of 0.05 to 125 ng/ml, and final concentrations were reported in femtomoles per million cells units.

### Drug quantification in pigtail macaque vaginal fluids

Mucosal fluid collected onto Merocel sponges was eluted by adding 2 ml of 70:30 ACN:water to 15-ml Falcon tubes containing a single sponge, vortexing for 1 to 2 min and aliquoting into a 1.5-ml microcentrifuge tube. Following protein precipitation extraction with ^13^C^15^N_3_-ISL, analytes were separated by using reverse-phase chromatography via a Waters Atlantis T3 (2.1 mm by 50 mm, 3.0-μm particle size) analytical column for macaque vaginal fluids. Precision and accuracy of the calibration standards and quality control samples were within 20% for the following dynamic range: 10 to 50,000 ng/ml of ISL in macaque vaginal fluid.

### Drug quantification in pigtail macaque vaginal tissues

Weighed tissues were transferred into Precellys hard tissue reinforced metal bead kit tubes (Cayman Chemical Company) containing 1 ml of 70:30 ACN:water, homogenized, and then centrifuged. Following protein precipitation extraction with ^13^C^15^N_3_-ISL and ^13^C_9_,^15^N_3_-dATP, ISL was separated by using reverse-phase chromatography via a Waters Atlantis T3 (2.1 mm by 50 mm, 3.0-μm particle size) analytical column, and ISL-TP was separated by using anion exchange chromatography via a Thermo Biobasic AX (2.1 mm by 50 mm, 5-μm particle size) analytical column. An AB Sciex API-5000 triple quadruple mass spectrometer was used to detect analytes and internal standards under positive ion electrospray conditions. Precision and accuracy of the calibration standards and quality control samples were within 20% for the following dynamic ranges: 0.1 to 500 ng/ml of ISL in macaque vaginal tissue and 0.1 to 100 ng/ml of ISL-TP in macaque vaginal tissue. Final concentrations were normalized to tissue mass and reported in nanograms per gram (ISL) or femtomoles per gram (ISL-TP) units.

### Macaque efficacy study

On the basis of the promising data from the 90-day PK study, we initiated a vaginal SHIV challenge study with a 60-mg IVR in sexually mature, normal cycling pigtail macaques. Six ISL IVR-treated animals received once weekly atraumatic low-dose SHIV162p3 vaginal inoculations, starting 3 days after IVR insertion, and then every 7 days thereafter for a total of 12 exposures. The presence of IVRs was visually confirmed 3 days after the fourth and eighth virus challenges to avoid tissue trauma on days of virus exposure. Two untreated, naïve real-time controls received the same weekly challenges. The rings were removed on day 90, and the macaques were followed for 10 weeks. Blood was collected throughout the study and processed to yield plasma and PBMCs. Samples were stored at −80°C, and drug concentrations in plasma (ISL) and PBMCs (ISL-TP) were determined at the end of the study. Plasma viremia and multicolor flow cytometry evaluation was carried out in real time throughout the study. We monitored the infection status by a quantitative real-time reverse transcription polymerase chain reaction (RT-PCR) assay as described previously (*58*, *62*, *69*). The RT-PCR detection limit was 50 copies/ml. Positive macaques were defined as having two consecutive PCR results above the detection limit. To account for the eclipse phase, we defined the time of infection as that from the challenge 7 days before SHIV RNA detection in plasma. SHIV serologic responses were qualitatively monitored by using a synthetic peptide enzyme immunoassay (GS HIV-1/HIV-2 PLUS O EIA, Bio-Rad, Hercules, CA). The macaques were considered protected if they remained SHIV RNA negative and seronegative during the 12 weeks of challenges and the 9.5-week follow-up period after IVR removal.

### Residual drug quantification post-IVR removal from pigtail macaques

Following completion of the 30-day PK, 90-day PK, and 90-day efficacy studies, IVRs were removed from pigtail macaques and extracted to quantify residual ISL post–in vivo release. Each IVR was weighed, and metrics (OD and CSD) were measured before extraction. The IVRs were subsequently placed in 50 ml of acetone for 48 hours to fully extract residual ISL from each ring. Extraction samples were collected and analyzed via HPLC to quantify the amount of drug remaining in the IVR. To determine the amount of ISL delivered over the study duration, the residual drug was subtracted from the known initial dose of ISL loaded into the IVR.

### Statistical methods

Statistics for changes in frequencies for the different CD4 and CD8 T cell subsets while the ISL IVR was present in the (i) 28-day PK (days 3 to 28 combined as one group), (ii) 90-day PK (days 3 to 90 combined as one group), and (iii) efficacy (weeks 1 to 13 combined as one group) in comparison to before ring insertion (day 0) and post-IVR removal (day 30/day 92/weeks 14 to 22 combined as one group for the 28-day, 90-day, and efficacy studies, respectively) was carried out with the Kruskal-Wallis test with Dunn’s posttest for multiple comparisons of the log0transformed values. This analysis was carried out for each animal individually during the efficacy phase. For all statistical tests, a *P* value of <0.05 was considered significant.
