# Supplementary material for: Dose-ranging pharmacokinetics, safety, and efficacy of a 90-day intravaginal ring delivering islatravir in pigtail macaques
Source: Sci Adv. 2026 Aug 26;12(35):eaef3514. doi: 10.1126/sciadv.aef3514 (PMC13510676; doi:10.1126/sciadv.aef3514)
Supplement: Supplementary file 1 [file sciadv.aef3514_sm.pdf]

Science Advances
AAAS

Supplementary Materials for

Dose-ranging pharmacokinetics, safety, and efficacy of a 90-day intravaginal ring delivering islatravir in pigtail macaques

Priya Srinivasan *et al.*

Corresponding author: J. Gerardo García-Lerma, jng5@cdc.gov;
Soumya Rahima Benhabbour, benhabs@email.unc.edu

Sci. Adv. **12**, eaef3514 (2026)
DOI: 10.1126/sciadv.aef3514

This PDF file includes:

Figs. S1 to S8
Tables S1 and S2

Supplementary Materials

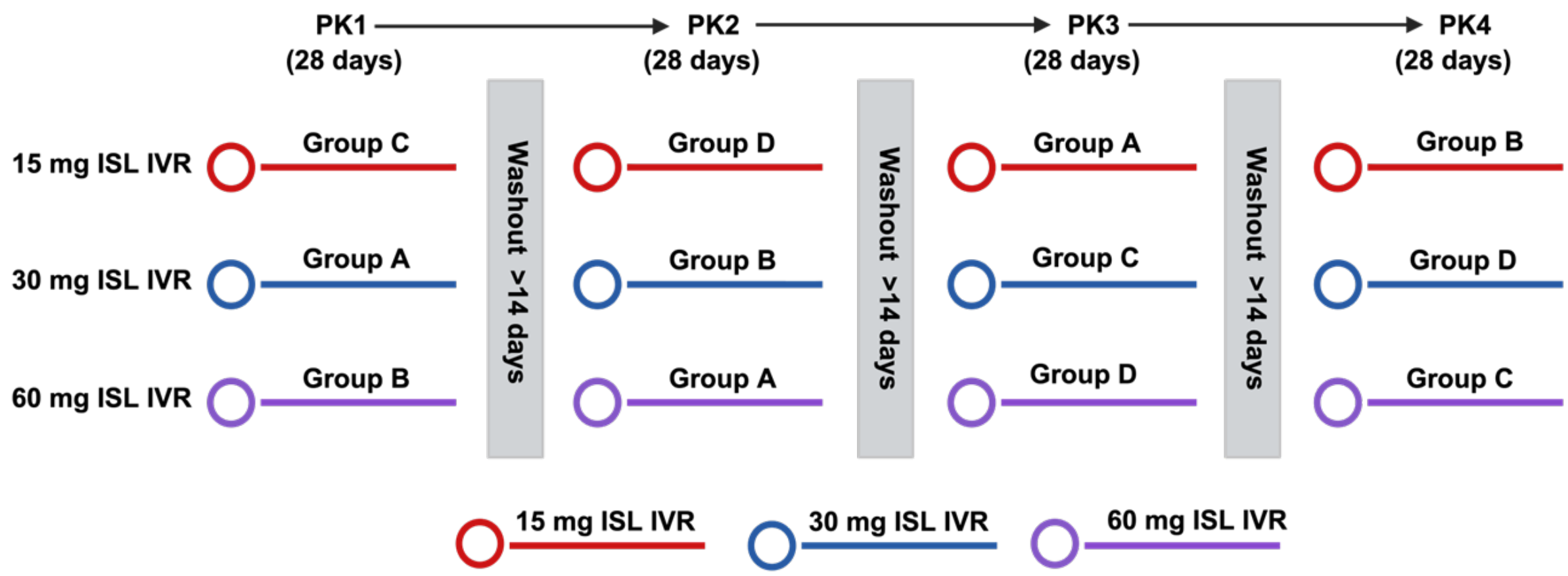

Fig. S1. 28-day PK cross-over study design. Eight pigtailed macaques were randomized into four groups (A, B, C, D) of n=2 each and scheduled for crossover PK studies as depicted. All eight animals received all three ISL doses for 28 days with washout periods of at least two weeks between each round.

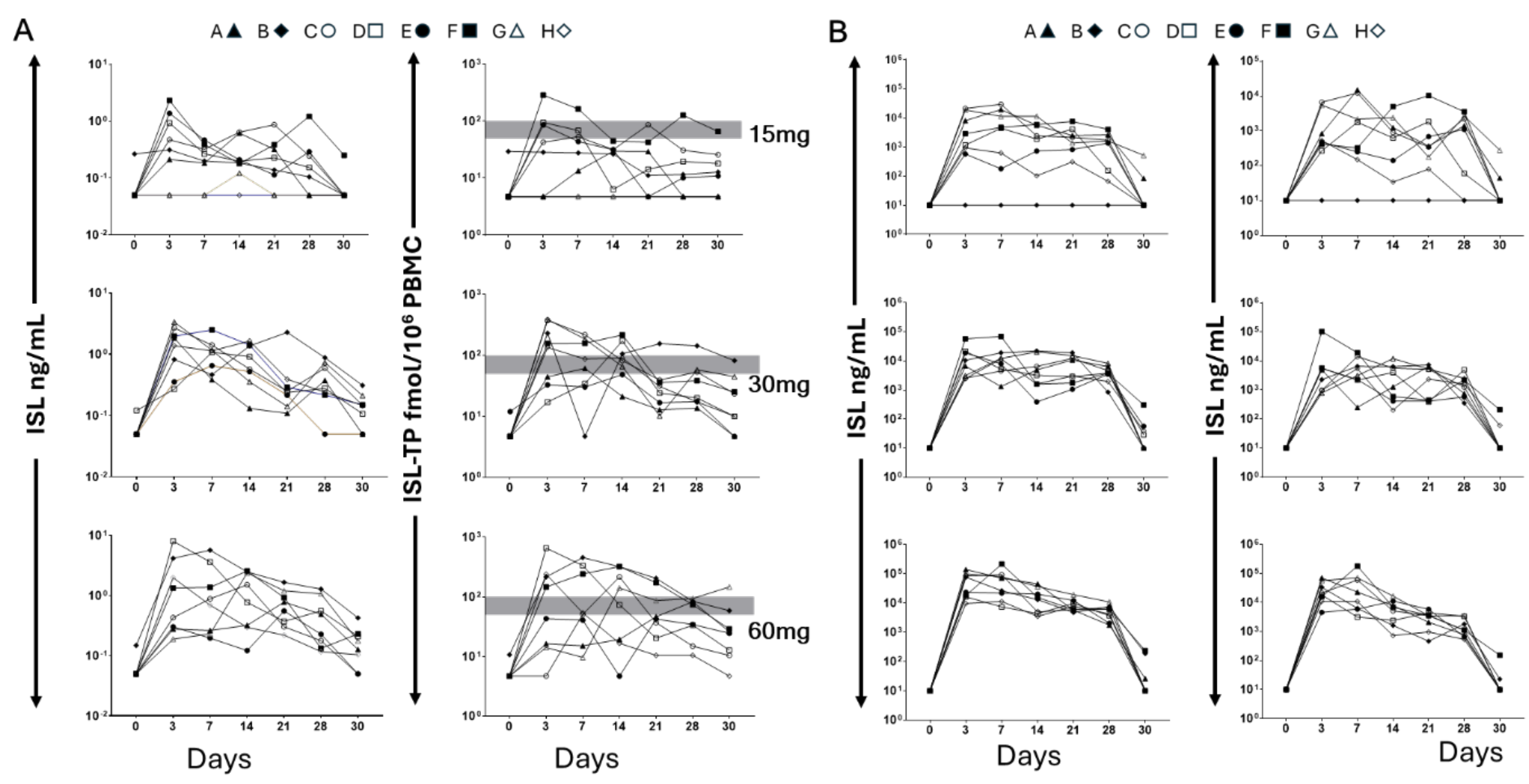

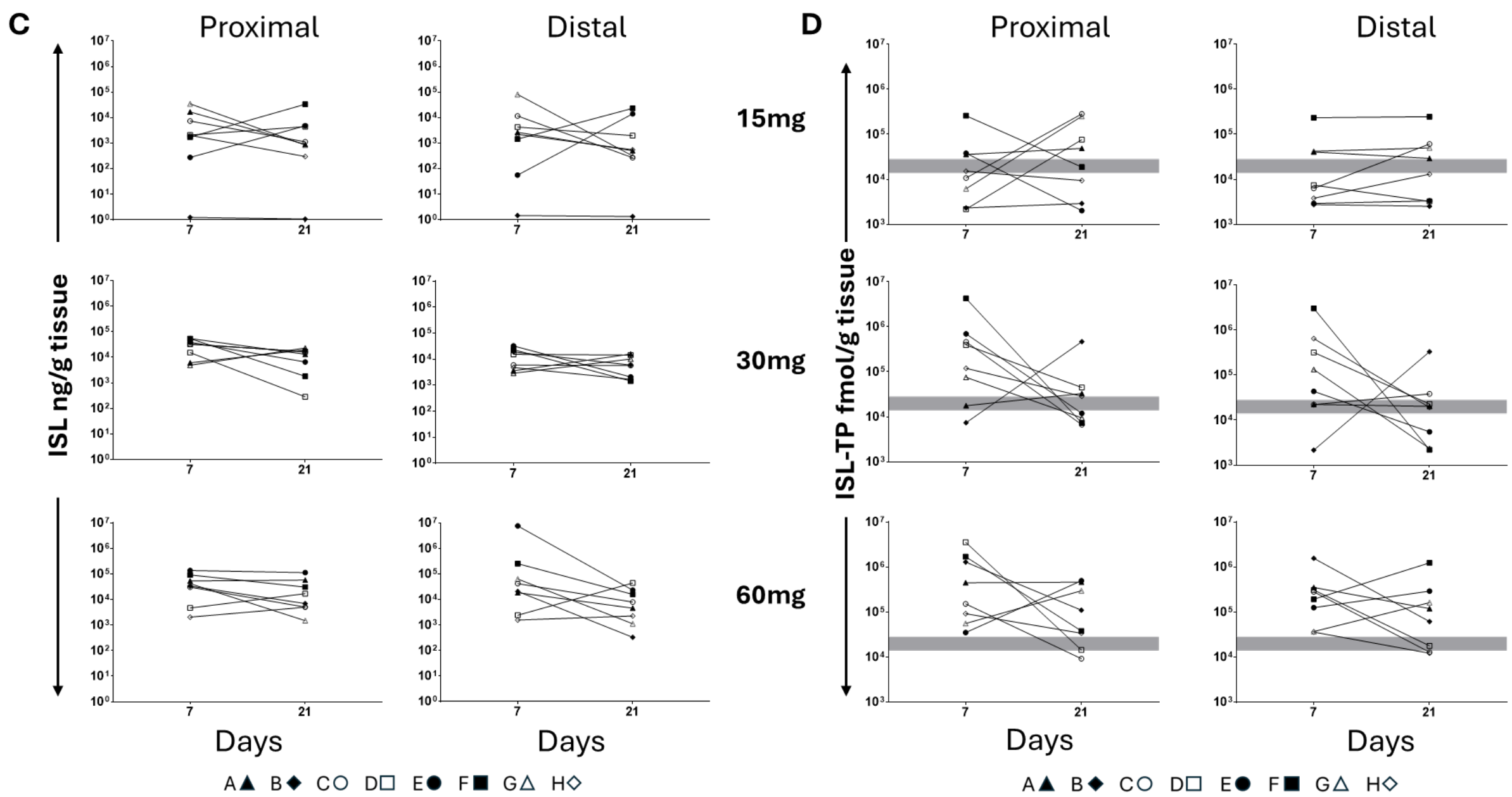

Fig. S2. 28-day PK individual macaque drug concentrations. Samples collected from eight pigtail macaques (macaque IDs A-H) were assayed for islatravir (ISL) and ISL-triphosphate (TP) concentration in (**A**) plasma (ng/mL), peripheral blood mononuclear cells (PBMCs, fmol/10^6 PBMC), (**B**) vaginal fluids (proximal and distal, ng/spear), (**C**) vaginal tissue (proximal, and distal, ng/g), and (**D**) vaginal tissue (proximal and distal, fmol/g). The gray bar in (A) denotes PK benchmark of 50–100 fmol/10^6 PBMCs and (D) converted to fmol/g of tissue based on a conversion factor of 284,355 cells/mg of tissue. All animals received all 3 doses for 28 days with washout periods between applications.

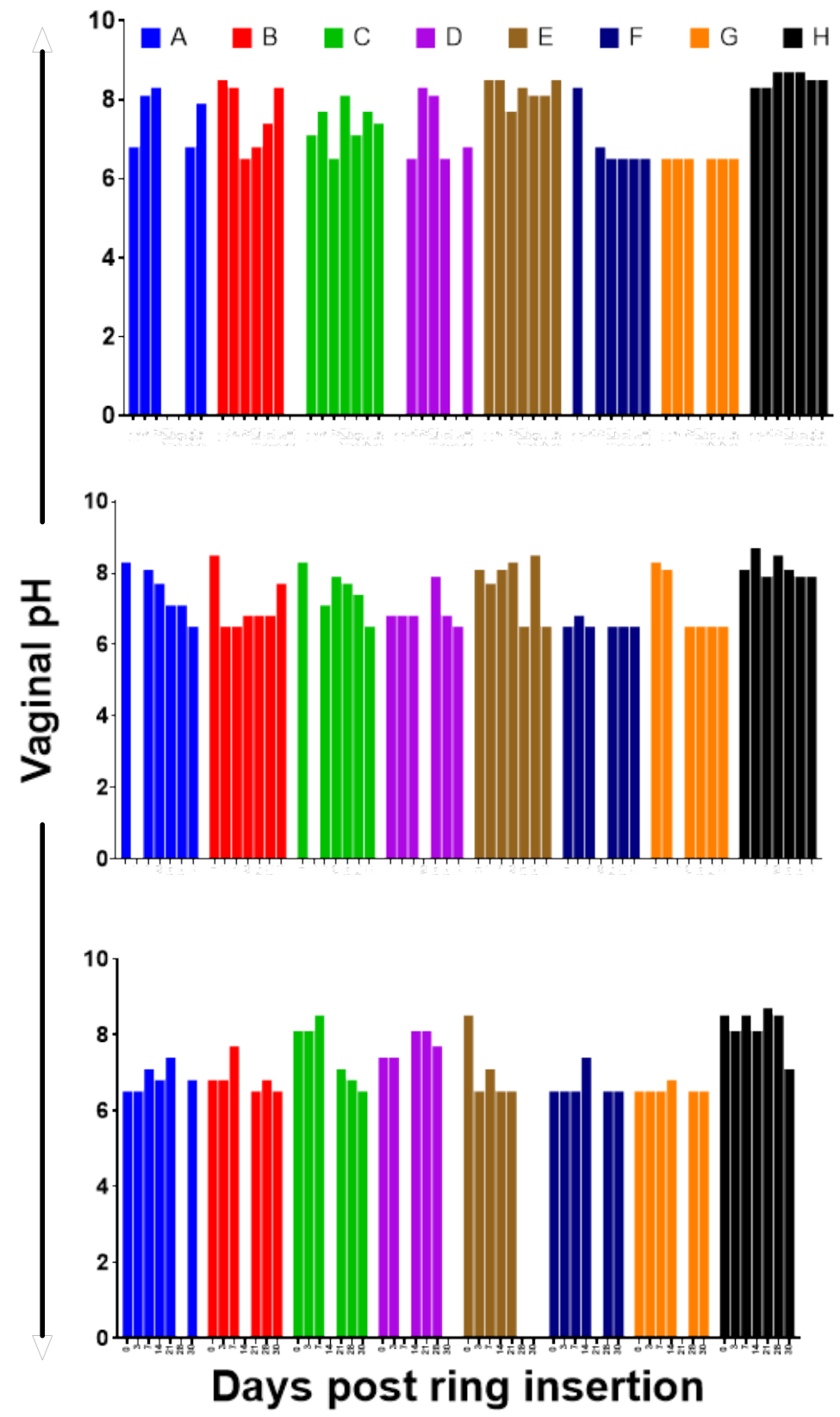

Fig. S3. Individual macaques' vaginal pH throughout the 28-day PK study. Individual macaques' vaginal pH during use of the 15 mg, 30 mg, and 60 mg IVRs in the 28-day PK study.

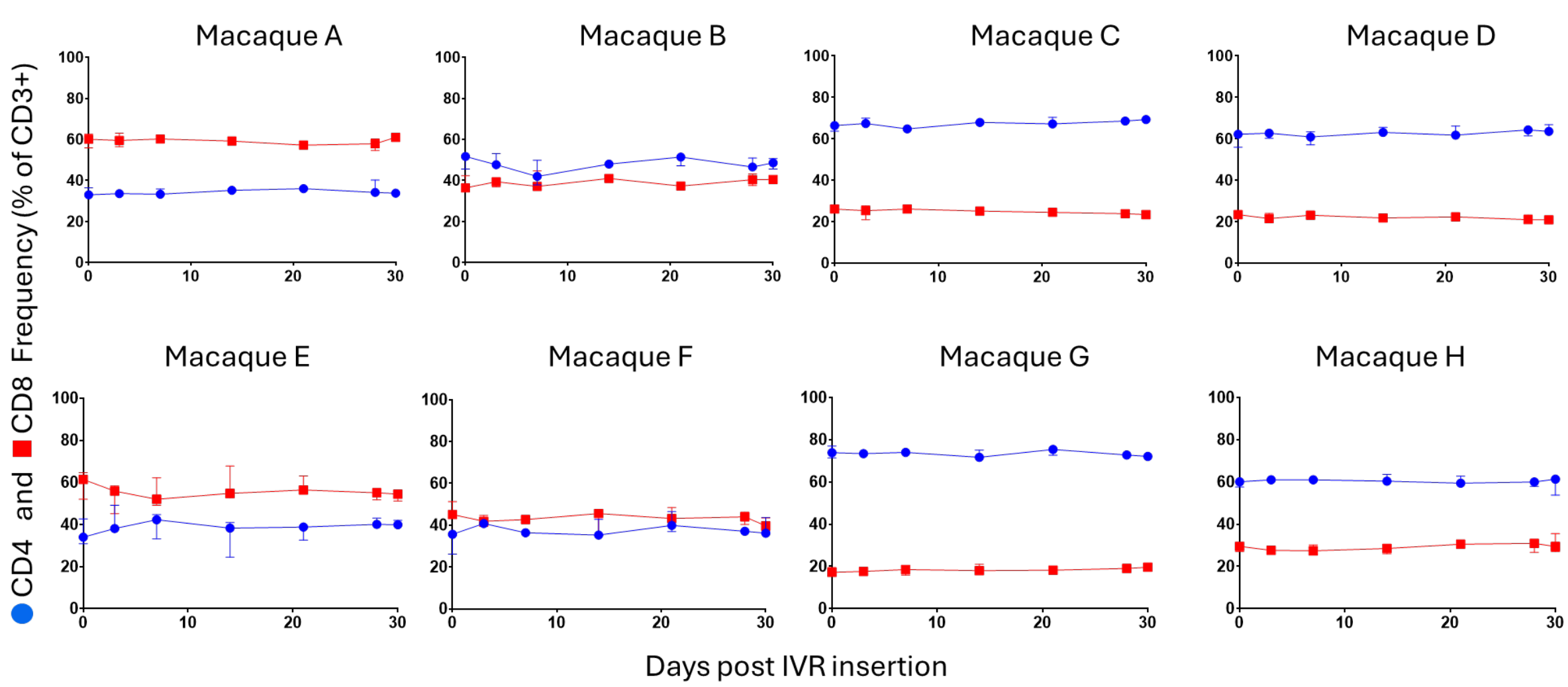

Fig. S4. 28-day PK—Individual macaques' T cell kinetics. Median and range of CD3+CD4+ and CD3+ CD8+ T-cell frequencies of individual macaques during use of the 15 mg, 30 mg, and 60 mg IVRs in the 28-day PK study.

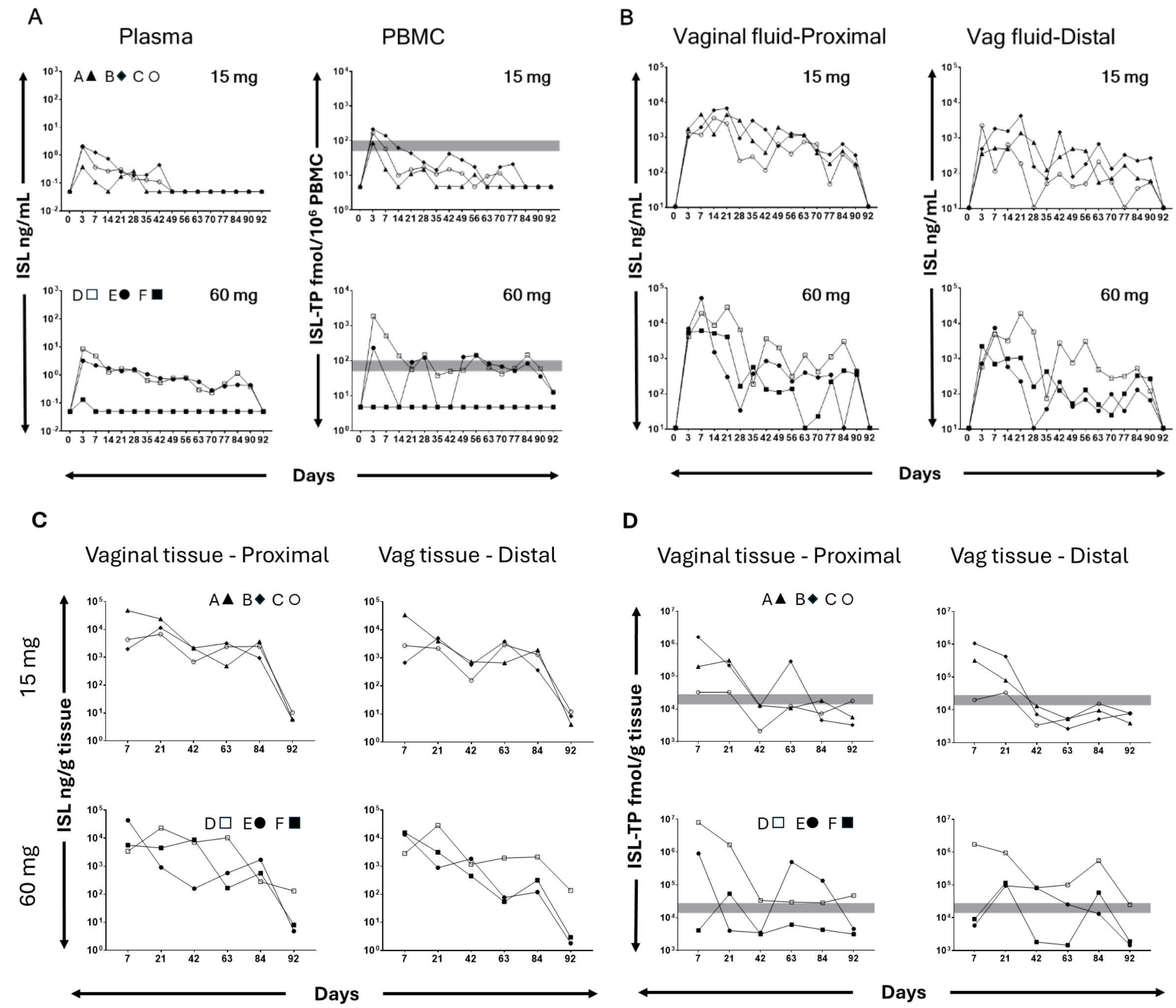

Fig. S5: 90-day PK individual macaque drug concentrations. Three pigtail macaques islatravir (ISL) and ISL-triphosphate (TP) concentration in (**A**) plasma (ng/mL), peripheral blood mononuclear cells (PBMCs, fmol/10^6 PBMCs), (**B**) vaginal fluid (proximal and distal, ng/spear), (**C**) vaginal tissue (proximal and distal, ng/g), and (**D**) vaginal tissue (fmol/g). All six macaques had the rings in place for 90 days. The gray bar in (A) denotes PK benchmark of 50–100 fmol/10^6

PBMCs and (D) converted to fmol/g of tissue based on a conversion factor of 284,355 cells/mg of tissue.

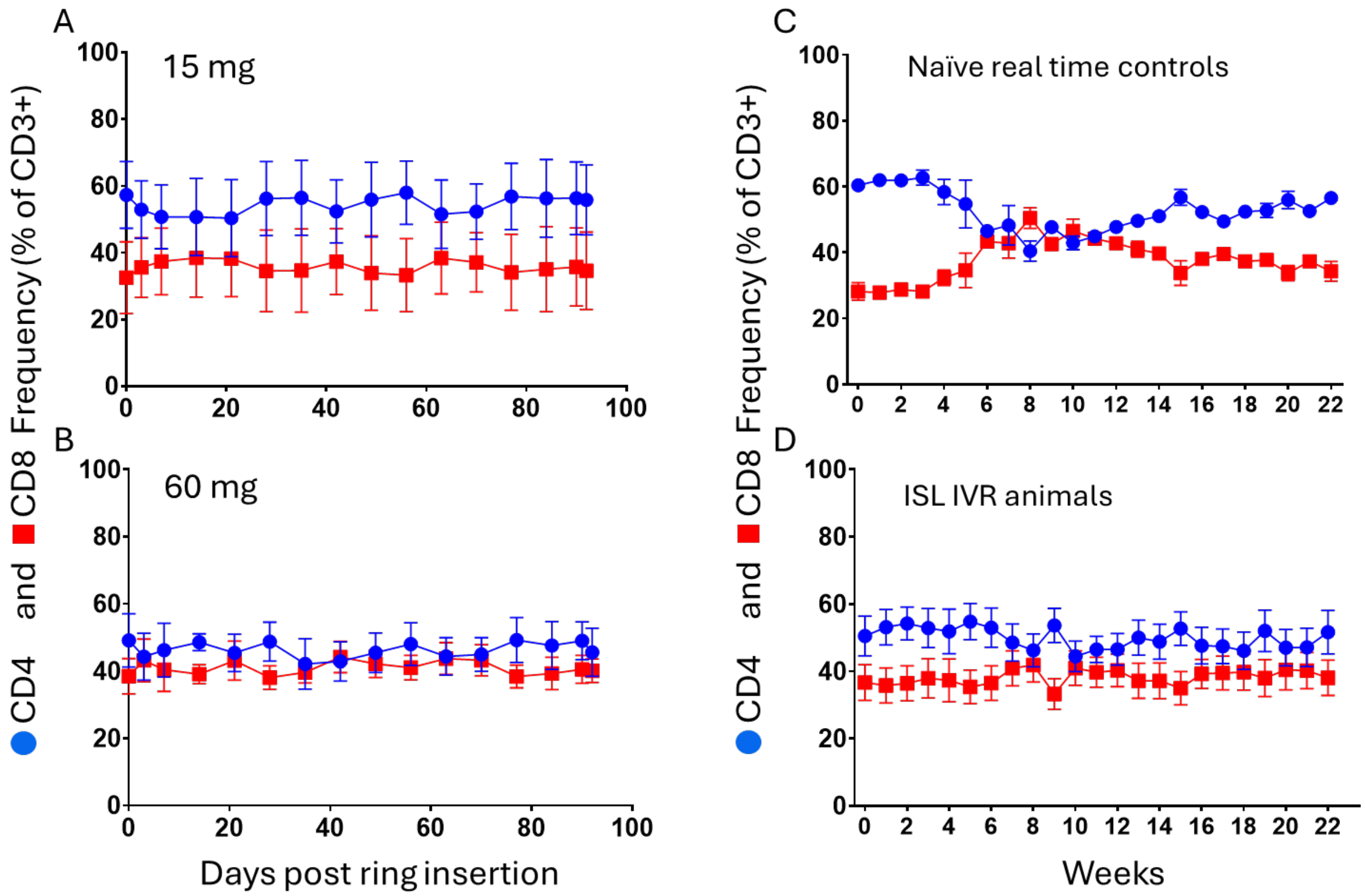

Fig. S6. 90-day PK T cell kinetics. Mean and standard error mean (SEM) of CD3+CD4+ and CD3+ CD8+ T cell frequencies of macaques that were administered (**A**) 15 mg ISL (n=3) and (**B**) 60 mg ISL (n=3) IVRs during the 90-day PK study, (**C**) naïve real-time controls, and (**D**) 60 mg (n=6) IVRs during the efficacy.

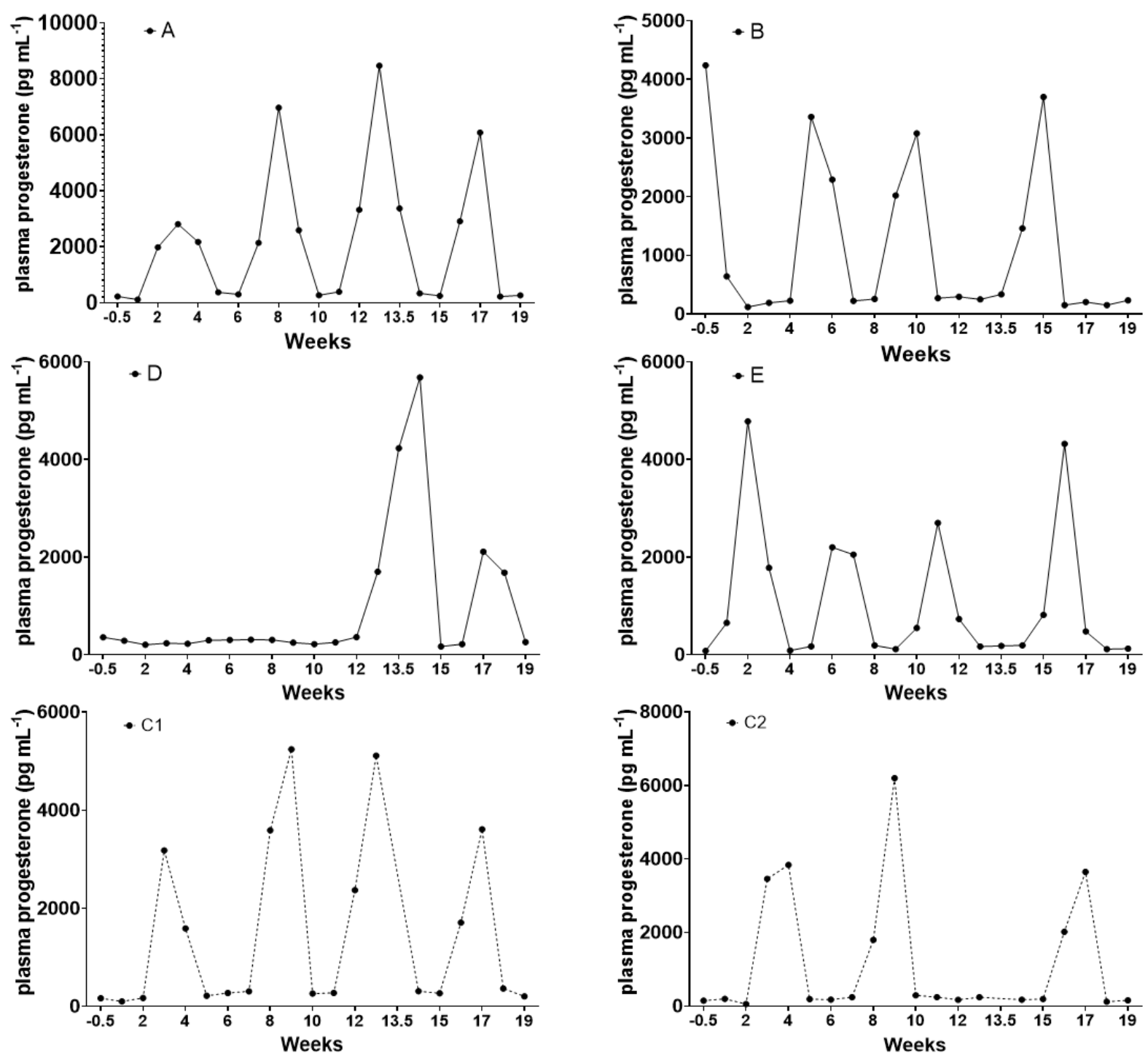

Fig. S7. 90-day efficacy individual macaques' plasma progesterone. Macaque plasma progesterone was monitored throughout the study to confirm normal cycles and determine timing of infection in reference to menstrual cycle. A–F, individual ISL IVR animals; C1 and C2, real-time naïve controls.

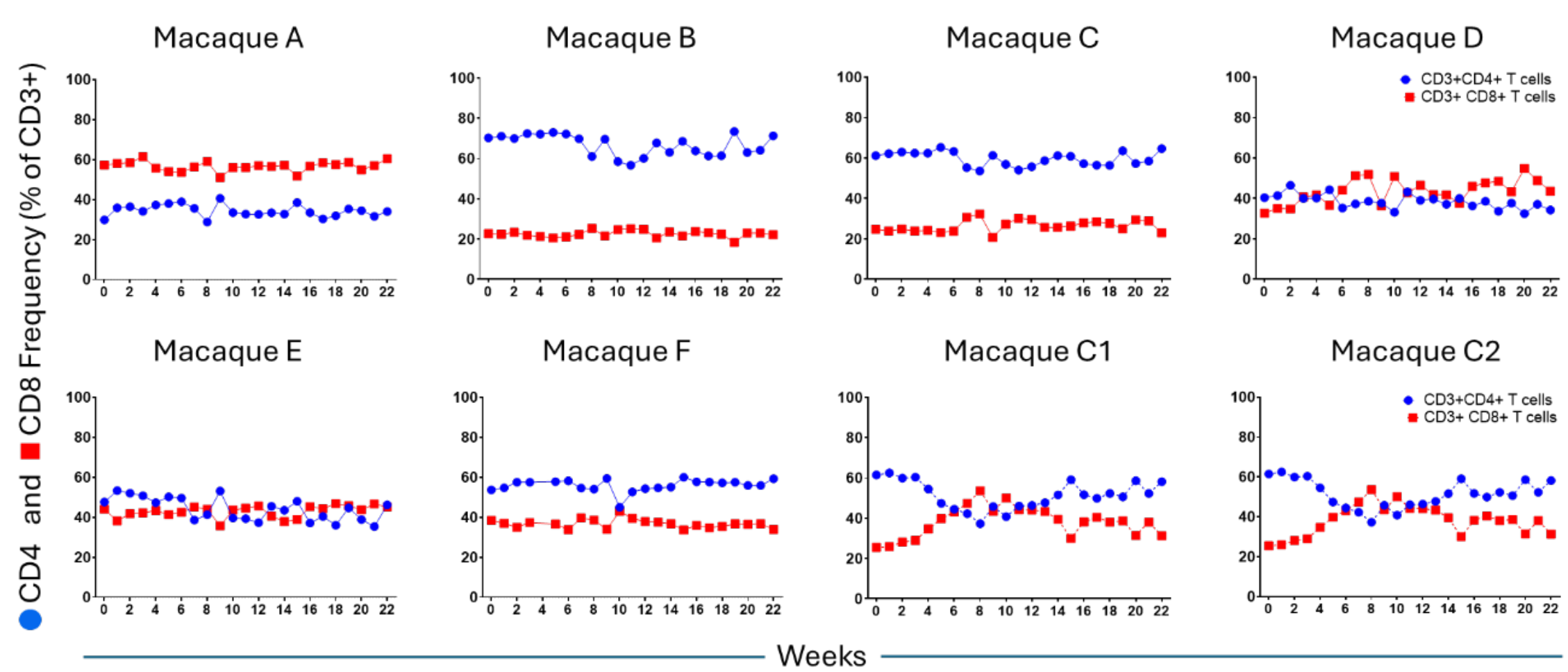

Fig. S8. 90-day efficacy individual macaques' T cell kinetics. CD4+ and CD3+ CD8+ T cell frequencies of untreated controls (n=2, C1 and C2) and 60 mg ISL IVR (n=6, A–F) macaques for 22 weeks.

Table S1: Summary table of median ISL and ISL-TP concentration in the different matrices.

Median (range) ISL and ISL-TP concentrations in plasma, PBMC, and vaginal fluid (Days 3-28)

Dose	Plasma (ISL- ng/mL)		PBMC (ISL-TP fmol/10^6 cells)		Vaginal fluid Proximal (ISL- ng/spear)		Vaginal fluid Distal (ISL-ng/spear)	
15 mg	**0.29**	(0.11-2.32)	**31.33**	(6.27-285.18)	**2,444.28**	(67.21-29,762.60)	**865.66**	(34.09-15,028.17)
30 mg	**0.60**	(0.11-3.36)	**53.66**	(10.23-392.12)	**5,102.81**	(390.60-70,075.50)	**2,317.30**	(201.17-103,511.52)
60 mg	**0.63**	(0.12-8.01)	**63.62**	(9.64-656.66)	**11,808.67**	(1,763.95-216,393)	**4,846.13**	(464.95-179,681.50)

Median (range) ISL and ISL-TP concentrations in vaginal tissue (Days 3-28)

Dose	Vaginal tissue Proximal (ISL- ng/g)		Vaginal tissue Distal (ISL- ng/g)		Vaginal tissue Proximal (ISL-TP- fmol/g)		Vaginal tissue Distal (ISL-TP- fmol/g)	
15 mg	**2,051.99**	(276.2-34,604.68)	**2,083.28**	(56.38-80,838.07)	**38,098.01**	(2,175-284,251.97)	**42,167.79**	(6,311.21-246,649.88)
30 mg	**16,544.21**	(282.62-54,326.66)	**5,919.03**	(1,441.33-32,290.50)	**38,845.67**	(6,672.64-4,246,690.52)	**38,281.96**	(5,501.20-3,044,998.52)
60 mg	**30,723.29**	(1,494.23-137,620.48)	**17,192.76**	(327.86-7,623,847.83)	**130,319.53**	(9,194.87-3,551,574.28)	**143,368.29**	(12,103.42-1,561,875.34)

Table S2: Summary table of median ISL and ISL-TP concentration in the different matrices.

Median (range) ISL and ISL-TP concentrations in plasma, PBMC, and vaginal fluid (Days 3-90)

Dose	Plasma (ISL- ng/mL)		PBMC (ISL-TP fmol/10^6 cells)		Vaginal fluid Proximal (ISL- ng/spear)		Vaginal fluid Distal (ISL-ng/spear)	
15 mg	**0.27**	(0.11-2.03)	**17.23**	(9.53-213.88)	**868.92**	(45.58-6,738.49)	**199.03**	(37.36-4,264.85)
60 mg	**0.76**	(0.13-8.45)	**82.18**	(35.27-1,848.03)	**513.18**	(23.27-52,227.50)	**278.01**	(25.50-19,008.46)

Median (range) ISL and ISL-TP concentrations in vaginal tissue (Days 3-90)

Dose	Vaginal tissue Proximal (ISL- ng/g)		Vaginal tissue Distal (ISL- ng/g)		Vaginal tissue Proximal (ISL-TP- fmol/g)		Vaginal tissue Distal (ISL-TP- fmol/g)	
15 mg	**2,475.46**	(482.88-48,035.62)	**1,895.12**	(157.60-33,646.62)	**25,415.31**	(4,533.50-1,618,154.23)	**14,242.52**	(2,658.50-1,071, 262.36)
60 mg	**3,458.14**	(159.90-43,626.04)	**1,845.43**	(54.29-28,813.07)	**53,557.09**	(4,287.50-8,000,041.69)	**87,941.63**	(1,453.50-1,722,105.68)
